# Traditional Chinese Medicine Intervenes Ventricular Remodeling Following Acute Myocardial Infarction: Evidence From 40 Random Controlled Trials With 3,659 Subjects

**DOI:** 10.3389/fphar.2021.707394

**Published:** 2021-08-31

**Authors:** Xiao-Xiao Zhang, Bo Liang, Chang-Le Shao, Ning Gu

**Affiliations:** ^1^ Nanjing University of Chinese Medicine, Nanjing, China; ^2^ Xuzhou Hospital of Traditional Chinese Medicine, Xuzhou, China; ^3^ Nanjing Hospital of Chinese Medicine Affiliated to Nanjing University of Chinese Medicine, Nanjing, China

**Keywords:** traditional Chinese medicine, acute myocardial infarction, ventricular remodeling, meta-analysis, randomized controlled trial

## Abstract

**Objectives:** We intend to conduct a meta-analysis on the systematic evaluation of traditional Chinese medicine (TCM) in the treatment of ventricular remodeling following acute myocardial infarction (AMI). Our findings may provide certain references for the clinical treatment of ventricular remodeling.

**Methods:** A systematic literature search was conducted in PubMed, Web of Science, Cochrane Library, Embase, CNKI, Wanfang Data, CQVIP, and CBM before 20 July 2020. Data were analyzed using a random/fixed-effect model. Primary outcomes included the effectiveness and TCM syndrome score (TCMSS). Secondary outcomes included 1) echocardiography data, including the left ventricular end-diastolic diameter (LVEDD), left ventricular end-systolic diameter (LVESD), left ventricular end-diastolic volume index (LVEDVi), left ventricular end-systolic volume index (LVESVi), left ventricular end-diastolic volume (LVEDV), left ventricular end-systolic volume (LVESV), interventricular septum thickness (IVST), left ventricular posterior wall thickness (LVPWT), left ventricular ejection fraction (LVEF), E/A, stroke volume (SV), and wall motion score (WMS); 2) serum indicators, including the B-type natriuretic peptide (BNP) or N-terminal pro-B-type natriuretic peptide (NT-proBNP), and C-reactive protein (CRP) or high sensitivity CRP (hs-CRP); (3) major adverse cardiovascular events (MACE) and other adverse events

**Results:** Forty RCTs involving 3,659 subjects were recruited. Our findings proved that a combination of TCM or TCM preparations with conventional Western medicine for preventing and reversing ventricular remodeling at post-AMI could remarkably enhance the total effectiveness and reduced TCMSS. Moreover, myocardial functions (LVEF, E/A, and SV), ventricular remodeling (LVEDVi, LVESVi, LVEDV, LVESV, LVEDD, LVESD, LVPWT, and WMS), serum levels of BNP and CRP, and MACE were significantly improved by the combination of TCM or TCM preparations with conventional Western medicine. Nevertheless, IVST and the incidence of other adverse events were comparable between control and experimental groups

**Conclusion:** The combination of TCM or TCM preparations and conventional Western medicine can alleviate the process of ventricular remodeling, enhance cardiac function, and reduce the incidence of MACE in AMI patients.

## Introduction

With the emergence and gradual application of percutaneous coronary intervention, the mortality due to acute myocardial infarction (AMI) has sharply declined (Bajaj et al., 2015). The hospitalization rate of AMI shows a decreasing trend in recent years. Nevertheless, there are 550,000 new onsets and 200,000 recurrent cases of AMI annually in the United States (Dariush et al., 2016). More seriously, the global burden of cardiovascular diseases and AMI mainly concentrates on low- and middle-income countries. At present, more than 80% of deaths from cardiovascular diseases occur in these countries (Murray et al., 2012; Murray et al., 2015). After AMI, due to oxidative stress, inflammatory response, neuroendocrine regulation, and other reasons, the morphological structure of the myocardium and cardiac fibroblasts changed significantly (Zhang et al., 2021). A series of pathological changes, in turn, affect ventricular size, structure, and function, that is, ventricular remodeling (Liang et al., 2020b). In fact, reduced cardiomyocytes and poor development of viable cardiomyocytes and extracellular matrix are the chief criminals of ventricular remodeling. They not only trigger adverse cardiac events but also activate multiple-system functions like the neurohormonal pathway due to the declined systolic function (McMurray, 2005). As a consequence, a vicious circle following AMI further aggravates ventricular remodeling, thereafter leading to the deterioration of cardiac function, heart failure, malignant arrhythmia, and even cardiac death.

Most drugs that delay ventricular remodeling exert their functions by antagonizing the renin-angiotensin-aldosterone system (RAAS) or cross-talking with it, including angiotensin-converting enzyme inhibitors (ACEIs), angiotensin II receptor blockers (ARBs) and aldosterone antagonists, and β-adrenergic receptor antagonists. Long-term combination therapy can improve clinical symptoms, hemodynamic status, and clinical outcomes of ventricular remodeling. However, ACEIs and ARBs can increase plasma renin activity, which only temporarily reduces plasma aldosterone levels, but cannot block mineralocorticoid receptors. The DREAM trial found that the inhibitory effect of ACEIs alone on the RAAS is far from enough. About 50% of patients with chronic heart failure would suffer from the escape of ACE inhibition (Roig et al., 2000). Although ARBs block the effect of AngII on AT1R, a large dose of ARBs can cause AngII accumulation in the body, which can directly activate AT2R. Although AT2R can antagonize some adverse effects of AT1R, overactivation of AT2R can also increase the risk of cardiovascular events by activating inflammatory factors or other pathological conditions. Mineralocorticoid receptor antagonists are not effective on some non-gene cardiovascular effects of aldosterone. Novel drugs for more effectively regulating the RAAS are under development, including the direct renin inhibitors, ACE2 agonists, AT2R agonists, and aldosterone synthase inhibitors. The direct renin inhibitor aliskiren has been clinically applied, the additional use of which with conventional drugs of ACEIs/ARBs and β-blockers in high-risk myocardial infarction with low left ventricular function cannot improve ventricular remodeling but even induce more adverse events like hypotension and increased serum creatinine and hyperkalemia. Therefore, aliskiren is not recommended to be additionally used in these patients (Solomon et al., 2011). Data of the ASTRONAUT trial demonstrated that the use of aliskiren in patients with chronic heart failure cannot reduce cardiovascular mortality and rehospitalization rate due to heart failure (Gheorghiade et al., 2013). Therefore, searching for effective adjuvant therapy for the current treatment of ventricular remodeling after myocardial infarction has become a new insight.

Compound medicine is superb in achieving a comprehensive outcome, rather than a single target. It contains multiple compounds or compound groups, aiming to obtain a better therapeutic efficacy through several pharmacological mechanisms (Luo et al., 2009). In particular, compound medicine has been extensively applied in the adjuvant treatment of cardiovascular diseases alongside Western medicine, which is featured by satisfactory outcomes and fewer adverse events (Liang et al., 2020a; Liang et al., 2020d). To our knowledge, compound medicine targeting ventricular remodeling lacks in the market. Traditional Chinese medicine (TCM) is natural and has a long history, which is becoming popular throughout the world. Most TCMs are compound preparations. Even a single substance drug has complex ingredients, rather than a single chemical composition. Under the guidance of TCM theory, TCM preparations (such as tablets, injections, aerosols, pills, powders, and pastes) have been widely used in the prevention and health management of human diseases owing to their unique advantages of multi-target involvement.

There is abundant *in vitro* and *in vivo* evidence on TCM treatment of ventricular remodeling following AMI. Chen et al. suggested that the Tongguan capsule obviously ameliorates ventricular remodeling and cardiac function in AMI rats, which is superior to captopril (Chen et al., 2010). In a Sprague-Dawley rat model of myocardial infarction, Danhong injection induces upregulation of fibrosis-associated genes (MMP-2 and MMP-9) and VEGF, as well as downregulation of caspase-3. In addition, vascular density in the infarct margin increased in rats administrated with Danhong injection, proving that Danhong injection effectively protects ventricular remodeling and cardiac function (Chen et al., 2016). Liang et al. demonstrated that the Qiliqiangxin capsule improves cardiac function and ventricular remodeling in heart failure rats at post-AMI via upregulating VEGF and phosphorylating Akt (Liang et al., 2016). However, it is noteworthy that a systematic evaluation of the clinical safety and efficacy of TCM compounds in the treatment of ventricular remodeling is lacked because of the deficiency of multi-center, large sample data, which significantly limits their reliability and popularization in clinical practice. In the present study, we intend to conduct a meta-analysis on the systematic evaluation of TCM compounds in the treatment of ventricular remodeling following AMI. Our findings may provide certain references for the clinical treatment of ventricular remodeling.

## Material and Methods

### Literature Searching

Relevant literature published before July 20, 2020, was searched in PubMed, Web of Science, Cochrane Library, Embase, CNKI, Wanfang Data, CQVIP, and CBM. The following MeSH (medical subject headings) and free words were searched in the combination: myocardial infarction/acute myocardial infarction + ventricular remodeling/myocardial remodeling/left ventricular remodeling + traditional Chinese medicine/TCM/combination of Chinese and Western medicine/Chinese patent drug/natural medicine + randomized controlled trial/random distribution/random. In addition, relevant data in the Chinese Clinical Trial Registry (ChiCTR) and ClinicalTrails were manually searched to avoid any missing information.

### Inclusion Criteria

Inclusion and exclusion criteria of random controlled trials (RCTs) about oral administrations of TCM and TCM preparations for intervening AMI were developed according to the Cochrane Handbook for Systematic Reviews of Interventions (version 5.0). Relevant clinical RCTs conducted by either blinding, allocation concealment, or comparison with negative control or placebo were not limited by languages. Research elements should be complete, including research objective, study design, and statistical processing. Recruited AMI patients were not limited by age, gender, time of onset, and case origin. Control subjects were treated by conventional Western medication, including reperfusion treatment, nitrates, anticoagulants, β-receptor blockers, ACEI, and lipid-regulating drugs, and placebos were also included. Meanwhile, TCM or TCM preparations were additionally supplied in the experimental group. General treatments like recumbent and oxygen inhalation were applied in both groups.

### Exclusion Criteria

The following literature was excluded: 1) unclear diagnostic criteria or non-AMI patients; 2) subjects who had severe primary diseases like brain, liver, or kidney diseases; 3) repeated studies; 4) animal experiments, case reports, reviews, summarize experiences, or ineligible studies lacking the rigorism, main indicators, texts or pathological data; and 5) subjects in the control group who were treated with TCM or TCM preparations.

### Quality Assessment

The quality of recruited literature was independently assessed by two investigators using a risk bias assessment tool as the Cochrane Handbook for Systematic Reviews of Interventions (version 5.0) suggested. Six items were considered, including the method of randomized allocation, allocation concealment, blinding, the integrity of results, selective reporting results, and other resources of biases.

### Data Extraction

Study selection and data extraction were independently conducted by two investigators based on the searching strategy. At first, searched literature was initially screened for titles and abstracts and re-screened for texts. The following items were extracted from eligible studies, including authors, year, interventions, length of time, sex composition, average age, results, and adverse events. Any disagreement was solved by the third investigator.

### Outcomes

Primary outcomes included the effectiveness and TCM syndrome score (TCMSS). Secondary outcomes included 1) echocardiography data, including the left ventricular end-diastolic diameter (LVEDD), left ventricular end-systolic diameter (LVESD), left ventricular end-diastolic volume index (LVEDVi), left ventricular end-systolic volume index (LVESVi), left ventricular end-diastolic volume (LVEDV), left ventricular end-systolic volume (LVESV), interventricular septum thickness (IVST), left ventricular posterior wall thickness (LVPWT), left ventricular ejection fraction (LVEF), E/A, stroke volume (SV), and wall motion score (WMS); 2) serum indicators, including the B-type natriuretic peptide (BNP) or N-terminal pro-B-type natriuretic peptide (NT-proBNP), and C-reactive protein (CRP) or high sensitivity CRP (hs-CRP); and 3) major adverse cardiovascular events (MACE) and other adverse events.

### Statistical Analysis

Data processing was conducted using Revman (version 5.3) and Stata (version 14.0). Relative risk (RR) was presented as the effect of binary data, while mean deviation (MD) or standard mean deviation (SMD) were used as that of continuous data. 95% confidence interval (CI) was calculated. The heterogeneity was assessed by the Chi-square test (Liang et al., 2019). *I* ^2^ < 50% and *p* > 0.1 were considered no heterogeneity and a fixed-effect model was adopted; otherwise, a random-effect model was introduced (Liang et al., 2021). Potential causes of the heterogeneity were analyzed through subgroup analyses, L’Abbe graph, or radial plots. Sensitivity analysis was performed for assessing the result reliability, and a descriptive analysis was replaced if the heterogeneity was considered high. Funnel plots were depicted for assessing publication biases. A significant difference was set at *p* < 0.05.

## Results

### Searching and Selecting Eligible Literature

**FIGURE 1:**
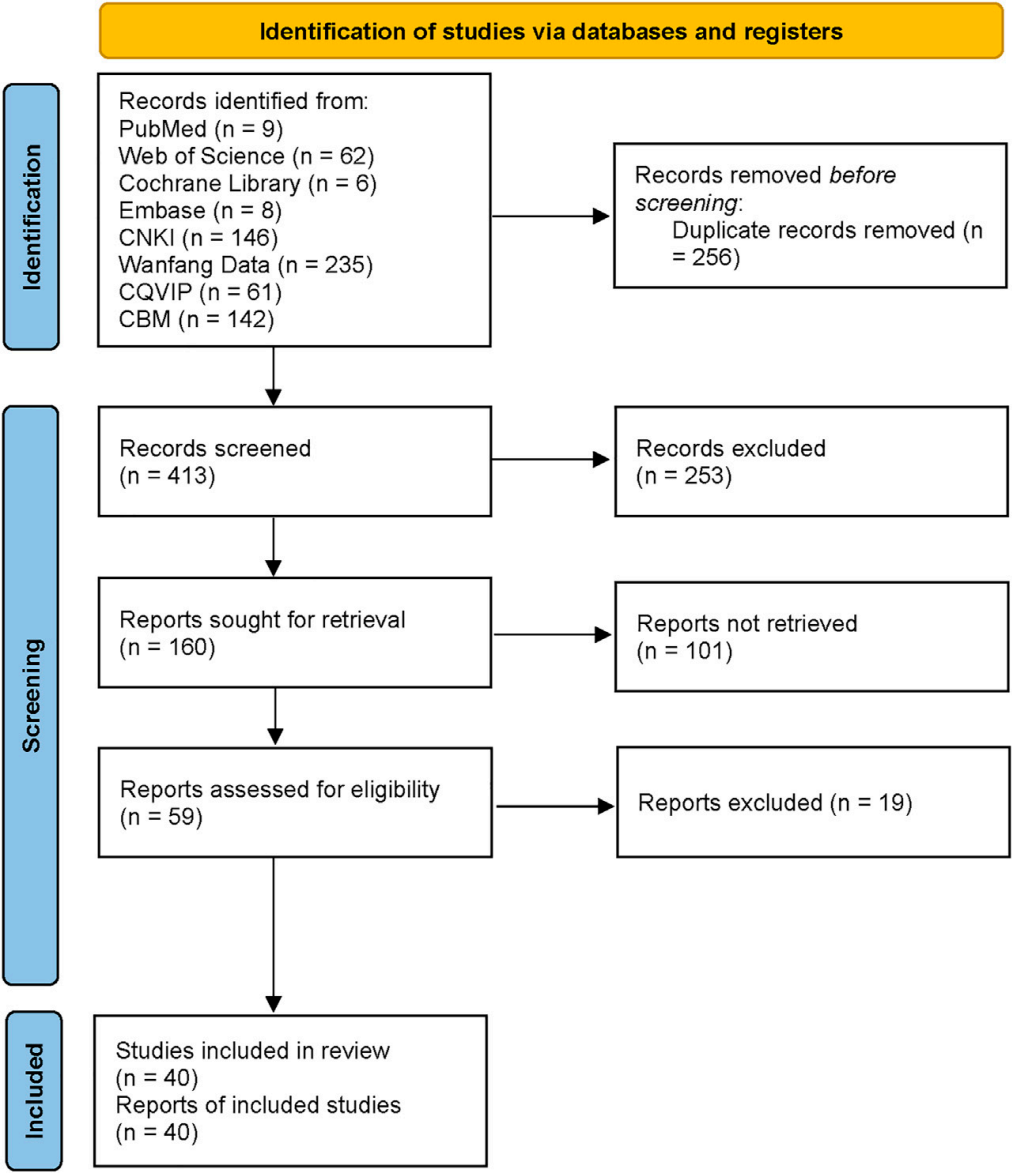
Flow diagraph of study selection.

At first, a total of 669 studies were initially searched. Endnote was used to manage the searched studies. After excluding repeated studies, 413 records were screened. 253 other-type studies were excluded, 160 eligible studies were recruited. Later, 101 studies were further excluded because of ineligible research contents or interventions. By reviewing texts in detail, 19 studies were excluded because they were not RCTs or the experiment was not rigorous. At last, 40 studies were recruited for meta-analysis (Figure 1).

### Characteristics of Recruited Studies

Among 40 recruited RCTs (Zhang et al., 2002a; Zhang et al., 2002b; Huang and Hong, 2004; Tang et al., 2004; Zhao et al., 2005; Chen et al., 2006; Dong, 2006; Feng et al., 2006; Zhang, 2006; Chao et al., 2007; Deng et al., 2007; Du et al., 2007; Li et al., 2007; Du et al., 2008; Zhang, 2008; Zhao, 2008; Lin, 2011; Ruan et al., 2011; Ruan, 2012; Yang et al., 2012; Fan, 2014; Li, 2014; Yang et al., 2014; Liu et al., 2016; Shuai et al., 2016; Gong et al., 2017; Jiang et al., 2017; Li, 2017; Wu, 2017; Fan, 2018; Huang et al., 2018; Kai et al., 2018; Li, 2018; Wang et al., 2018; Xiang et al., 2019; Xu et al., 2019; Fang, 2020; Jiang, 2020; Mao et al., 2020; Yang et al., 2020), there were 3,659 subjects, including 1,845 controls treated with conventional Western medicine and 1,814 subjects treated with Western medicine combined with TCM or TCM preparations. Their baseline characteristics are listed in Supplementary Table S1. The details of conventional Western medication are shown in Supplementary Table S2, and details of TCM are shown in Supplementary Table S3.

### Quality Assessment of Recruited Studies

A total of 25/40 studies reported the method of randomized allocation by random number table or software. The remaining only mentioned that subjects were randomly assigned. One study described allocation concealment in detail. There were four double-blinded studies, and others did not point out blinding or non-blinding (Supplementary Figure S1).

### Outcomes

#### Primary Outcomes

**FIGURE 2:**
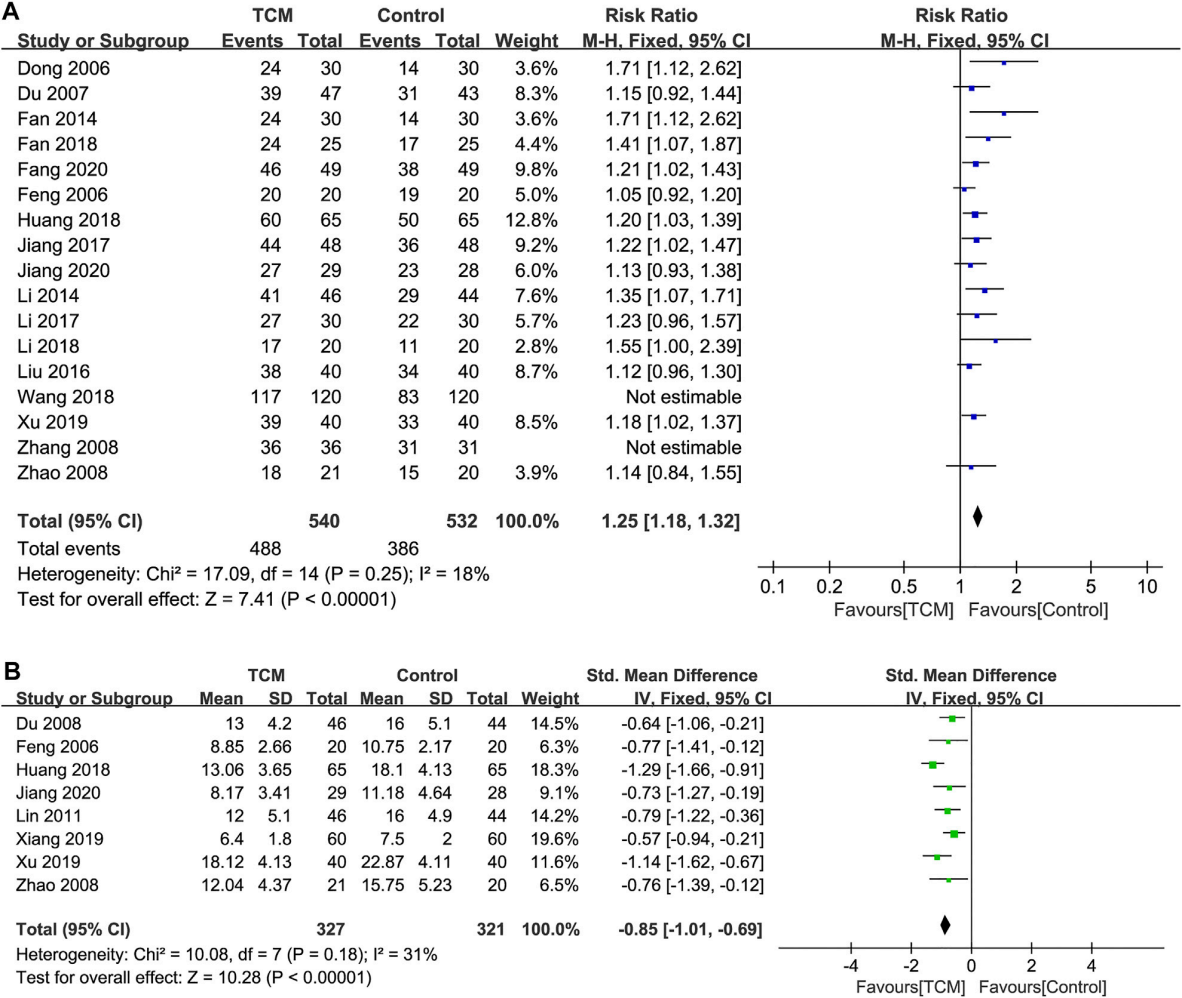
Primary outcomes. **(A)**. Effectiveness. **(B)**. TCMSS.

The heterogeneity of the selected 17 studies (Dong, 2006; Du et al., 2007; Fan, 2014; Fan, 2018; Fang, 2020; Feng et al., 2006; Huang et al., 2018; Jiang, 2020; Jiang et al., 2017; Li, 2018; Li, 2017; Li, 2014; Liu et al., 2016; Wang et al., 2018; Xu et al., 2019; Zhang, 2008; Zhao, 2008) reporting the effectiveness of TCM interventions was considered high (*I* ^*2*^ = 80%, *p* = 0.00001 < 0.1), which could be attributed to studies conducted by Zhang 2008 (Zhang, 2008) and Wang 2018 (Wang et al., 2018) via the analysis of the L'Abbe plot (Supplementary Figure S2), radial plot (Supplementary Figure S3A), and sensitivity analysis (Supplementary Figure S4). After removing them, there was no heterogeneity in the remaining 15 studies (*I* ^*2*^ = 18%, *p* = 0.25), and thus, a fixed-effect model was adopted. RR of the 15 studies was 1.25 (95% CI = 1.18–1.32, Z = 7.41, *p* < 0.05), suggesting that the effectiveness of a combination of TCM or TCM preparations was superb than that of conventional Western medicine (Figure 2A).

**FIGURE 3:**
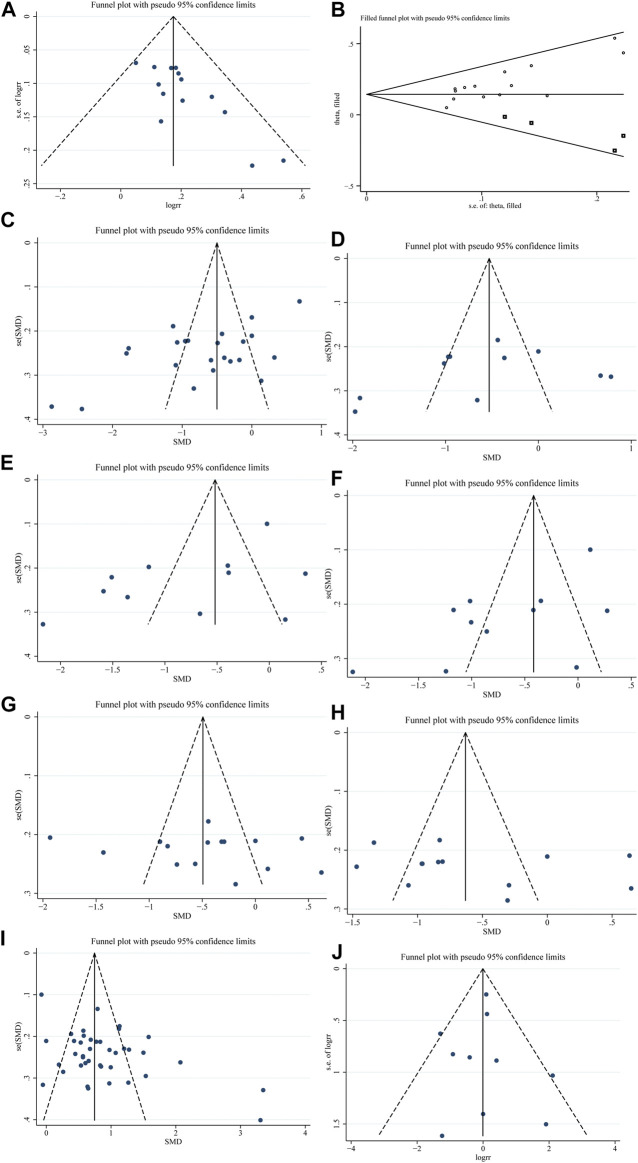
Funnel plots. **(A)**. Effectiveness. **(B)**. Effectiveness after trim. **(C)**. LVEDD. **(D)**. LVESD. **(E)**. LVEDVi. **(F)**. LVESVi. **(G)**. LVEDV. **(H)**. LVESV. **(I)**. LVEF. **(J)**. Other adverse events.

Funnel plots were depicted aiming to assess publication biases, and an asymmetric funnel graph suggested the absence of publication bias (Figure 3A). As Begg’s test shown, a certain publication bias existed in the 15 studies (*p* = 0.003). We thereafter processed the non-symmetric funnel plots through trim and filling. The four points of the square indicated the effectiveness of studies that should be recruited in the future. Taken into consideration of funnel plots, similar studies to Dong 2006 (Dong, 2006), Fan 2014 (Fan, 2014), Li 2018 (Wang et al., 2018), and Fan 2018 (Fan, 2018) are required to be recruited, aiming to eliminate the publication bias (Figure 3B). There was no heterogeneity in the eight studies (Du et al., 2008; Zhao, 2008; Lin, 2011; Huang et al., 2018; Xiang et al., 2019; Xu et al., 2019; Fang, 2020; Jiang, 2020) reporting TCMSS of TCM interventions (*I* ^2^ = 31%, *p* = 0.18), and as a result, a fixed-effect model was adopted. SMD of the eight studies was -0.85 (95% CI = -1.01 ∼ -0.69, *Z* = 10.28, *p* < 0.05), suggesting that a combination of TCM or TCM preparations was superb in reducing TCMSS than that of conventional Western medicine (Figure 2B). Since the sample size was less than 10, and we did not identify any heterogeneity, publication bias and sensitivity were unnecessary to be examined.

#### Second Outcomes

##### Echocardiography Findings

###### Indicators for Improving Ventricular Remodeling

####### Left Ventricular End-Diastolic Diameter

**FIGURE 4:**
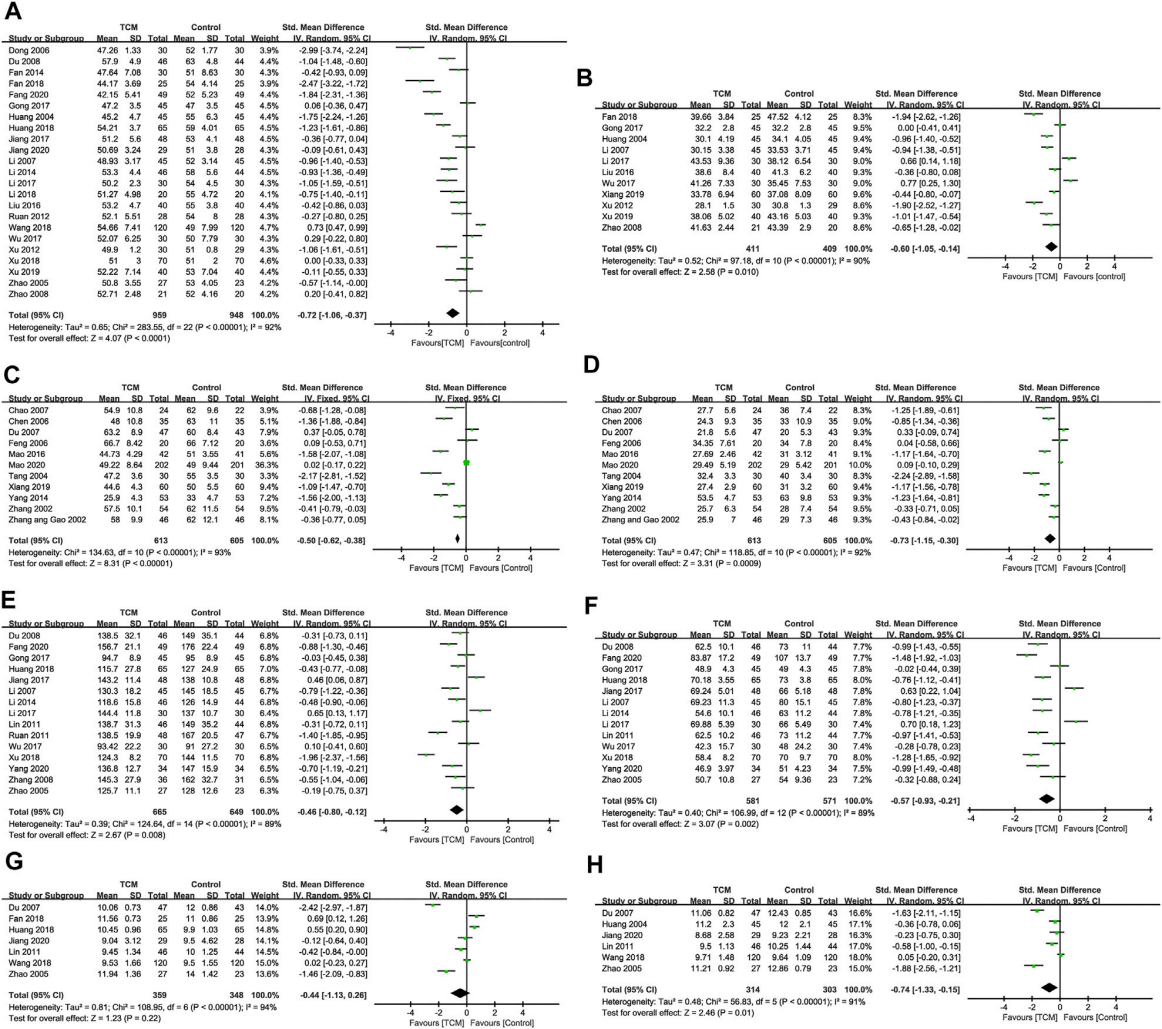
Indicators for improving ventricular remodeling. **(A)**. LVEDD. **(B)**. LVESD. **(C)**. LVEDVi. **(D)**. LVESVi. **(E)**. LVEDV. **(F)**. LVESV. **(G)**. IVST. **(H)**. LVPWT.

There was a significant heterogeneity among 23 studies (Huang and Hong, 2004; Zhao et al., 2005; Dong, 2006; Li et al., 2007; Du et al., 2008; Zhao, 2008; Ruan, 2012; Yang et al., 2012; Fan, 2014; Li, 2014; Liu et al., 2016; Gong et al., 2017; Jiang et al., 2017; Li, 2017; Wu, 2017; Fan, 2018; Huang et al., 2018; Kai et al., 2018; Li, 2018; Wang et al., 2018; Xu et al., 2019; Fang, 2020; Jiang, 2020) (*I* ^*2*^ = 92%, *p* < 0.1), and its source was analyzed by conducting radial plots and sensitivity analysis. Nevertheless, we failed to identify the source of the heterogeneity, which may be explained by differences in the course of interventions, examination instruments, and individualized experiences. As a result, a random-effect model was introduced for meta-analysis. SMD of the 23 studies was -0.72 (95% CI = -1.06 ∼ -0.37, *Z* = 4.07, *p* < 0.05), suggesting that a combination of TCM or TCM preparations was superb in reducing LVEDD than that of conventional Western medicine (Figure 4A). The funnel plots were symmetrically distributed (Begg’s test, *p* = 0.113), indicating the absence of a publication bias (Figure 3C).

####### Left Ventricular End-Systolic Diameter

There was a significant heterogeneity among 11 studies (*I* ^*2*^ = 90%, *p* < 0.1) (Huang and Hong, 2004; Li et al., 2007; Zhao, 2008; Yang et al., 2012; Liu et al., 2016; Gong et al., 2017; Li, 2017; Wu, 2017; Fan, 2018; Xiang et al., 2019; Xu et al., 2019), and we failed to identify the source of the heterogeneity. Therefore, a random-effect model was introduced for meta-analysis. SMD of the 11 studies was -0.60 (95% CI = -1.05 ∼ -0.14, Z = 2.58, *p* = 0.010 < 0.05), suggesting that a combination of TCM or TCM preparations was superb in reducing LVESD than that of conventional Western medicine (Figure 4B). The funnel plots were symmetrically distributed (Begg’s test, *p* = 0.436 > 0.05), indicating the absence of a publication bias (Figure 3D).

####### Left Ventricular End-Diastolic Volume Index

There was a significant heterogeneity among 11 studies (*I* ^*2*^ = 93%, *p* < 0.1) (Zhang et al., 2002a; Zhang et al., 2002b; Tang et al., 2004; Chen et al., 2006; Dong, 2006; Feng et al., 2006; Chao et al., 2007; Du et al., 2007; Yang et al., 2014; Shuai et al., 2016; Xiang et al., 2019; Mao et al., 2020), and we failed to identify the source of the heterogeneity. Therefore, a random-effect model was introduced for meta-analysis. SMD of the 11 studies was -0.78 (95% CI = −1.24 ∼ −0.32, *Z* = 3.32, *p =* 0.0009 < 0.05), suggesting that a combination of TCM or TCM preparations was superb in reducing LVEDVi than that of conventional Western medicine (Figure 4C). The funnel plots were symmetrically distributed (Begg’s test, *p* = 0.213 > 0.05), indicating the absence of a publication bias (Figure 3E).

####### Left Ventricular End-Systolic Volume Index

There was a significant heterogeneity among 11 studies (*I* ^*2*^ = 92%, *p* < 0.1) (Zhang et al., 2002a; Zhang et al., 2002b; Tang et al., 2004; Chen et al., 2006; Dong, 2006; Feng et al., 2006; Chao et al., 2007; Du et al., 2007; Yang et al., 2014; Shuai et al., 2016; Xiang et al., 2019; Mao et al., 2020), and we failed to identify the source of the heterogeneity. Therefore, a random-effect model was introduced for meta-analysis. SMD of the 11 studies was -0.73 (95% CI = −1.15 ∼ −0.30, *Z* = 3.31, *p =* 0.0009 < 0.05), suggesting that a combination of TCM or TCM preparations was superb in reducing LVESVi than that of conventional Western medicine (Figure 4D). The funnel plots were symmetrically distributed (Begg’s test, *p* = 0.276 > 0.05), indicating the absence of a publication bias (Figure 3F).

####### Left Ventricular End-Diastolic Volume

There was a significant heterogeneity among 15 studies (*I* ^*2*^ = 89%, *p* < 0.1) (Zhao et al., 2005; Li et al., 2007; Du et al., 2008; Zhang, 2008; Lin, 2011; Ruan et al., 2011; Li, 2014; Gong et al., 2017; Jiang et al., 2017; Li, 2017; Wu, 2017; Huang et al., 2018; Kai et al., 2018; Fang, 2020; Yang et al., 2020), and we failed to identify the source of the heterogeneity. Therefore, a random-effect model was introduced for meta-analysis. SMD of the 15 studies was -0.46 (95% CI = −0.80 ∼ −0.12, *Z* = 2.67, *p* = 0.008), suggesting that a combination of TCM or TCM preparations was superb in reducing LVEDV than that of conventional Western medicine (Figure 4E). The funnel plots were symmetrically distributed (Begg’s test, *p* = 0.843), indicating the absence of a publication bias (Figure 3G).

####### Left Ventricular End-Systolic Volume

There was a significant heterogeneity among 13 studies (*I* ^*2*^ = 89%, *p* < 0.1) (Zhao et al., 2005; Li et al., 2007; Du et al., 2008; Lin, 2011; Li, 2014; Gong et al., 2017; Jiang et al., 2017; Li, 2017; Wu, 2017; Huang et al., 2018; Kai et al., 2018; Fang, 2020; Yang et al., 2020), and we failed to identify the source of the heterogeneity. Therefore, a random-effect model was introduced for meta-analysis. SMD of the 13 studies was -0.57 (95% CI = −0.93 ∼ −0.21, *Z* = 3.07, *p =* 0.002 < 0.05), suggesting that a combination of TCM or TCM preparations was superb in reducing LVESV than that of conventional Western medicine (Figure 4F). The funnel plots were symmetrically distributed (Begg’s test, *p* = 0.951), indicating the absence of a publication bias (Figure 3H).

####### Interventricular Septum Thickness

There was a significant heterogeneity among seven studies (*I* ^*2*^ = 89%, *p* < 0.1) (Zhao et al., 2005; Du et al., 2007; Lin, 2011; Fan, 2018; Huang et al., 2018; Wang et al., 2018; Jiang, 2020), and we failed to identify the source of the heterogeneity. Therefore, a random-effect model was introduced for meta-analysis. SMD of the seven studies was -0.44 (95% CI = −1.13–0.26, *Z* = 1.23, *p =* 0.22 > 0.05), suggesting that there was no significant difference in IVST between a combination of TCM or TCM preparations with conventional Western medicine (Figure 4G).

####### Left Ventricular Posterior Wall Thickness

There was a significant heterogeneity among six studies (*I* ^*2*^ = 91%, *p* < 0.1) (Huang and Hong, 2004; Zhao et al., 2005; Du et al., 2007; Lin, 2011; Wang et al., 2018; Jiang, 2020), and we failed to identify the source of the heterogeneity. Therefore, a random-effect model was introduced for meta-analysis. SMD of the six studies was -0.74 (95% CI = −1.33 ∼ −0.15, *Z* = 2.46, *p =* 0.01), suggesting that a combination of TCM or TCM preparations was superb in reducing LVPWT than that of conventional Western medicine (Figure 4H).

###### Indicators for Improving Cardiac Function

####### Left Ventricular Ejection Fraction

**FIGURE 5:**
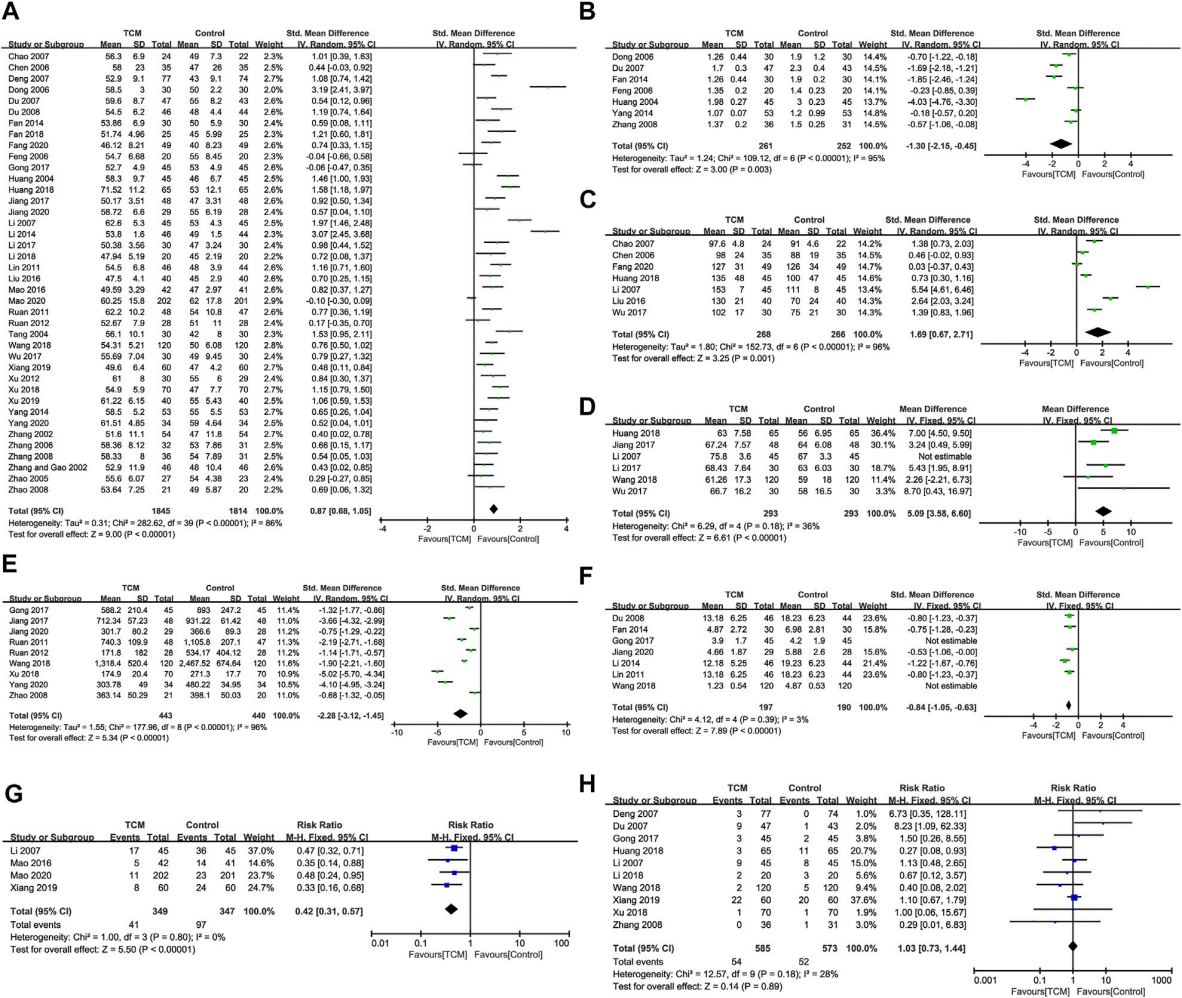
Indicators for improving cardiac function, serum indicators, and MACE. **(A)**. LVEF. **(B)**. WMS. **(C)**. E/A. **(D)**. SV. **(E)**. BNP/NT-pro BNP. **(F)**. CRP/hs-CRP. **(G)**. MACE. **(H)**. Other adverse events.

There was a significant heterogeneity among all 40 studies (*I* ^*2*^ = 86%, *p* < 0.1), and we failed to identify the source of the heterogeneity. Therefore, a random-effect model was introduced for meta-analysis. SMD of the 40 studies was 0.87 (95% CI = 0.68–1.05, *Z* = 9.00, *p* < 0.05), suggesting that a combination of TCM or TCM preparations was superb in LVEF than that of conventional Western medicine (Figure 5A). The funnel plots were symmetrically distributed (Begg’s test, *p* = 0.213), indicating the absence of a publication bias (Figure 3I).

####### Wall Motion Score

There was a significant heterogeneity among seven studies (*I* ^*2*^ = 95%, *p* < 0.1) (Huang and Hong, 2004; Dong, 2006; Feng et al., 2006; Du et al., 2007; Zhang, 2008; Fan, 2014; Yang et al., 2014), and we failed to identify the source of the heterogeneity. Therefore, a random-effect model was introduced for meta-analysis. SMD of the seven studies was -1.30 (95% CI = −1.06 ∼ −0.08, *Z* = 3.00, *p = 0.003* < 0.05), suggesting that a combination of TCM or TCM preparations was superb in reducing WMS than that of conventional Western medicine (Figure 5B).

####### E/A

There was a significant heterogeneity among seven studies (*I* ^*2*^ = 96%, *p* < 0.1) (Feng et al., 2006; Chao et al., 2007; Deng et al., 2007; Li et al., 2007; Liu et al., 2016; Wu, 2017; Huang et al., 2018), and we failed to identify the source of the heterogeneity. Therefore, a random-effect model was introduced for meta-analysis. SMD of the seven studies was 1.69 (95% CI = 0.83–1.96, *Z* = 3.25, *p =* 0.001 < 0.05), suggesting that a combination of TCM or TCM preparations was superb in E/A than that of conventional Western medicine (Figure 5C).

####### Stroke Volume

The heterogeneity of the selected six studies reporting the SV of TCM interventions was considered high (*I* ^*2*^ = 73%, *p* = 0.002) (Li et al., 2007; Jiang et al., 2017; Li, 2017; Wu, 2017; Huang et al., 2018; Wang et al., 2018), which could be attributed to studies conducted by Li 2007 (Li et al., 2007) (Supplementary Figure S3, S18D). After removing it, there was no heterogeneity in the remaining five studies (*I* ^*2*^ = 36%, *p* = 0.18), and thus a fixed-effect model was adopted. MD of the five studies was 5.09 (95% CI = 3.58–6.60, Z = 6.61, *p* < 0.05), suggesting that the effectiveness of a combination of TCM or TCM preparations was superb in SV than that of conventional Western medicine (Figure 5D).

###### Serum Indicators

There was a significant heterogeneity among nine studies (*I* ^*2*^ = 96%, *p* < 0.1) (Gong et al., 2017; Jiang, 2020; Jiang et al., 2017; Kai et al., 2018; Ruan et al., 2011; Ruan, 2012; Wang et al., 2018; Yang et al., 2020; Zhao, 2008), and its source was analyzed by conducting radial plots, sensitivity analysis, and subgroup analysis. Nevertheless, we failed to identify the source of the heterogeneity, which may be explained by differences in age, gender, course, and commercial kits. As a result, a random-effect model was introduced for meta-analysis. SMD of the nine studies was -2.28 (95% CI = −1.32 ∼ −0.05, *Z* = 5.34, *p* < 0.05), suggesting that a combination of TCM or TCM preparations was superb in reducing BNP/NT-pro BNP than that of conventional Western medicine (Figure 5E). There was a significant heterogeneity among seven studies (*I* ^*2*^ = 98%, *p* < 0.1) (Du et al., 2008; Lin, 2011; Fan, 2014; Li, 2014; Gong et al., 2017; Wang et al., 2018; Jiang, 2020), which could be attributed to studies conducted by Wang 2018 (Wang et al., 2018) and Gong 2017 (Gong et al., 2017) (Supplementary Figure S3C). After removing them, there was no heterogeneity in the remaining five studies (*I* ^*2*^ = 3%, *p* = 0.39), and thus a fixed-effect model was adopted. SMD of the five studies was −0.84 (95% CI = −1.05 ∼ −0.63, *Z* = 7.89, *p* < 0.05), suggesting that a combination of TCM or TCM preparations was superb in reducing CRP/hs-CRP than that of conventional Western medicine (Figure 5F).

###### Major Adverse Cardiovascular Events and Other Adverse Events

There was no heterogeneity in the four studies (Li et al., 2007; Mao et al., 2020; Shuai et al., 2016; Xiang et al., 2019) reporting MACE of TCM interventions (*I* ^*2*^ = 0, *p* = 0.80 > 0.1), and as a result, a fixed-effect model was adopted. RR of the four studies was 0.42 (95% CI = 0.31–0.57, *Z* = 5.50, *p* < 0.05), suggesting that a combination of TCM or TCM preparations was superb in MACE than that of conventional Western medicine (Figure 5G). There was no heterogeneity among the 10 studies (*I* ^*2*^ = 28%, *p* = 0.18 > 0.1) (Deng et al., 2007; Du et al., 2007; Li et al., 2007; Zhang, 2008; Gong et al., 2017; Huang et al., 2018; Kai et al., 2018; Li, 2018; Wang et al., 2018; Xiang et al., 2019). Therefore, a fixed-effect model was introduced for meta-analysis. RR of the 10 studies was 1.03 (95% CI = 0.73–1.44, *Z* = 0.14, *p =* 0.89 > 0.05), suggesting that there was no significant difference in other adverse events between a combination of TCM or TCM preparations with conventional Western medicine (Figure 5H). The funnel plots were symmetrically distributed (Begg’s test, *p* = 0.721), indicating the absence of a publication bias (Figure 3J).

### Sensitivity Analysis

The heterogeneity of relevant studies reporting the effectiveness was remarkably improved after removing studies conducted by Zhang 2008 (Zhang, 2008) and Wang 2018 (Wang et al., 2018). Echocardiography data (LVEDSi, LVEDVi, LVEDV, LVESD, LVEDD, LVESE, IVST, LVPWT, LVEF, E/A, and WMS) presented a high heterogeneity and its source was unable to be identified after a series of analyses, which may be attributed to differences in age, gender, course, instrument, and experience of the operator. In particular, the heterogeneity of relevant studies reporting SV was markedly improved after removing the study conducted by Li 2007 (Li et al., 2007). The heterogeneity source of BNP, however, was unknown, and we believed that age, gender, course, and commercial kits may cause the source. Studies conducted by Gong 2017 (Gong et al., 2017) and Wang 2018 (Wang et al., 2018) were the sources of the heterogeneity of CRP. Fixed-effect models were introduced for analyzing TCMSS, MACE, and adverse events since they did not have any heterogeneity.

Sensitivity analysis was performed for assessing the result reliability. Removal of any study did not obviously affect the general results, suggesting the robust results we obtained, except for effectiveness results of Zhang 2008 (Zhang, 2008) and Wang 2018 (Wang et al., 2018), SV results of Li 2007 (Li et al., 2007), and CRP/hs-CRP results of Gong 2017 (Gong et al., 2017) and Wang 2018 (Wang et al., 2018) (Supplementary Figures S4–S6). And the 4 RCTs were considered as the source analysis of heterogeneity of effectiveness, SV, and CRP/hs-CRP. We did not identify any heterogeneity about TCMSS, MACE, and other adverse events; therefore, sensitivity was unnecessary to be examined.

### Subgroup Analysis

The subgroup analysis was carried out when we try to find the heterogeneity of BNP/NT-proBNP and CRP/hs-CRP. However, there was nothing that could be found. We did not perform the subgroup analysis about other outcomes. In addition, we found that the duration of studies also varies, from weeks to months, so we conducted subgroup analysis according to the intervention duration. According to the results of intervention duration subgroup analysis (Supplementary Table S4), we found that in some outcome indicators, the heterogeneity was not completely eliminated after grouping, indicating that the difference of intervention duration may not be the potential source of heterogeneity.

## Discussion

At present, Western medicine is widely applied in the treatment and reverse of ventricular remodeling at post-AMI. For instance, imidapril, a type of ACEI, is able to suppress the remodeling of the left ventricle by inhibiting MMP activity in the plasma of AMI patients (Yokota et al., 2014). ARBs exert a similar function to that of ACEIs (Yokota et al., 2010). Statins like atorvastatin are demonstrated to defend inflammatory response via downregulating IL-1, IkBα, and NF-κB p50, thus preventing ventricular remodeling in rats (Karla et al., 2016). Besides, statin treatments are capable of alleviating ventricular remodeling in AMI patients through recovering the coronary endothelial function through endogenous nitric oxide (Ishida et al., 2012). The guidelines recommend that ACEI should be applied to all AMI patients as soon as possible, and ARB is recommended for patients who cannot tolerate ACEI. ACEI/ARB doses need to be titrated to the target dose or maximum tolerable dose. In the past 2 years, sacubitril/valsartan has been proven to be superior to ACEI/ARB in improving cardiac outcomes and reversing ventricular remodeling (Hajra et al., 2019). Besides, sacubitril/valsartan could improve exercise tolerance (Malfatto et al., 2020; Piepoli et al., 2021), showing promising applications in the treatment of AMI and reducing all-cause deaths. The TCM theory considers that blood stasis syndrome, phlegm-dampness syndrome, Qi-deficiency syndrome, Yang-deficiency syndrome, deficiency in origin, and excess in superficiality or deficiency-excess mixing occur following AMI. Therefore, TCM treatment mainly focuses on the holistic and individualized treatment of AMI patients. In clinical practice, *Pinellia ternata*, *Carthamus tinctorius*, Peach Kernel, *Salvia miltiorrhiza*, *Trichosanthes kirilowii*, *Scutellaria baicalensis*, *Codonopsis*, and Monkey Grass are usually used in the treatment of AMI. TCM compounds or preparations are composed of a single or multiple TCM under the guidance of the TCM principle and method, which are adjusted based on ancient, classical, and modern prescriptions (Luo et al., 2009). Generally speaking, Western medicine is featured by a direct function on the lesion and target, while TCM highlights the balance of the whole body (Liang et al., 2020c). It is considered that a combination of Western medicine and TCM is capable of enhancing the therapeutic efficacy through taking both of their advantages, thus achieving the goal of the simultaneous treatment of root and tip, rather than a single target/organ/system (Liang et al., 2020d). Recent evidence underlying the mechanisms of TCM in treating ventricular remodeling at post-AMI has emerged. It is reported that the Heart-Protecting Musk Pill treatment remarkably declines the collagen-positive area and improves the left ventricular function by downregulating IL-6 and TNF-α in rats following AMI (Cen et al., 2017). A combination of Tanshinone IIA and puerarin effectively inhibits infiltration of inflammatory cells, myocardial fibrosis, and ventricular fibrosis, therefore protecting the hemodynamics of AMI mice (Gao et al., 2019). As a result, the application of TCM or TCM preparations in the treatment of ventricular remodeling at post-AMI should be well concerned.

Once myocardial ischemia occurs, inflammatory factors (IL-1, caspase-1) immediately trigger the strong infiltration of neutrophils, and lymphocytes and macrophages are also involved in, that is, the inflammation phase (Mezzaroma et al., 2011; Yan et al., 2013). Later, the enlarged ischemia lesion acutely expands the ventricle and the infarcted ventricular wall. Meanwhile, extracellular matrixes are degraded and cardiac fibroblasts are rapidly proliferated, leading to the synthesis of collagens, that is, the proliferation phase. With the pathological process, the newly formed scar becomes mature which maintains the shape of the heart and prevents the formation of an aneurysm, that is, the mature phase (Frangogiannis, 2014; Seropian et al., 2014). In the initial phase, the heart is able to compensatively maintains the cardiac output through ventricular dilation. However, the compensatory mechanism eventually causes cardiac dysfunction and heart failure over time (Seropian et al., 2014). In clinical practice, echocardiography is generally applied to effectively reflect the degree of ventricular remodeling by quantifying the diameter, volume, and wall thickness of the left ventricle. In the present study, we comprehensively analyzed echocardiography data from 40 RCTs and demonstrated that a combination of TCM or TCM preparations with conventional Western medicine more pronouncedly reduced LVEDSI, LVEDVI, LVEDV, LVESV, LVEDD, LVESD, and LVPWT in AMI patients in comparison to controls (*p* < 0.05). IVST was comparable between groups (*p* > 0.05). BNP or NT-proBNP is a quantified biomarker of heart failure that refers to the diastolic and systolic functions of the left ventricle. It is usually utilized for assessing the prognosis of AMI (Alvin et al., 2013). Here, we assessed the prognosis of ventricular remodeling at post-AMI and MACE by detecting cardiac functions. It is shown that a combination of TCM or TCM preparations with conventional Western medicine was superb in elevating LVEF, E/A, and SV and reducing wall motion score, serum level of BNP, and MACE rate than controls (*p* < 0.05). In addition, the results of SV were robust after removing a single study conducted by Li 2007 (Li et al., 2007). Publication bias about LVEF was absent as well. CRP is an acute-phase protein that is produced by the stimulation of pro-inflammatory cytokines, which is considered a risk factor for cardiovascular diseases. Excessive inflammation will aggravate the occurrence of fibrosis and lead to pathological remodeling (Kaneko et al., 2011; Anzai, 2018; Rita et al., 2018) Our results revealed that combining treatment of TCM or TCM preparations with Western medicine more obviously reduced serum level of CRP in AMI patients than those who solely treated with Western medicine (*p* < 0.05). After removing studies of Gong 2017 (Gong et al., 2017) and Wang 2018 (Wang et al., 2018), the results remained robust. We did not obtain a significant difference in the rate of adverse events between groups (*p* > 0.05). The determination of the effectiveness and TCMSS was in accordance with the Guiding Principles for Clinical Research of New Chinese Medicines (Trial) in 2002, the Diagnostic Criteria of TCM Syndrome Differentiation for Coronary Heart disease revised by the Chinese Association of Integrative Medicine in October 1990 (Zhao, 2008), and Guidelines for the Diagnosis and Treatment of Acute Coronary Heart disease (CHD Myocardial Infarction) (Zhao, 2008). Compared with controls, TCM or TCM preparation combined with Western medicine markedly elevated the total effectiveness and reduced TCMSS (*p* < 0.05). After removing studies of Zhang 2008 (Zhang, 2008) and Wang 2018 (Wang et al., 2018), the single study posed few impacts on the total effectiveness. In addition, recruitment of similar studies to Dong 2006 (Dong, 2006), Fan 2014 (Fan, 2014), Li 2018 (Li, 2018), and Fan 2018 (Fan, 2018) in the future is able to eliminate the current publication bias.

Taken together, this study systematically assesses the function of TCM or TCM preparations in the treatment of vascular remodeling at post-AMI. A total of 40 RCTs, involving 3,659 AMI patients, were recruited. Our findings proved that a combination of TCM or TCM preparations with conventional Western medicine for preventing and reversing ventricular remodeling at post-AMI could remarkably enhance the total effectiveness and myocardial function (LVEF, E/A, SV, and WMS), manifesting as reduced TCMSS, the diameter, volume and wall thickness of the left ventricle (LVEDSi, LVEDVi, LVEDV, LVESV, LVEDD, LVESD, and LVPWT), serum levels of BNP and CRP, and MACE rate. Nevertheless, IVST and the incidence of other adverse events were comparable between control and experimental groups. Because of differences in age, sex, course, instruments, and individualized experiences, the heterogeneity of echocardiography data and serum levels of CRP and BNP are considered high, and its source should be further determined by subgroup analyses. Although TCM is considered to be safe because of no significant difference in the incidence of other adverse events between groups, only 10 studies mentioned adverse events during the treatment. Therefore, the safety of TCM or TCM preparation in the treatment of ventricular remodeling at post-AMI requires to be further validated in clinical trials. This study for the first time assessed that the additional use of TCM or TCM preparations based on conventional Western medicine significantly improved ventricular remodeling at post-AMI, which were conductive to make clinical decisions.

## Limitations

Nevertheless, the present study has some limitations. First of all, TCM is mainly applied in China. As a result, relevant clinical data abroad are lacked. Secondly, the quality of recruited literature should be concerned as the specific randomized method and blinding are not mentioned in some studies. Thirdly, mortality is rarely reported in the recruited studies. Hence, we are unable to determine the long-term efficacy of TCM and its further clinical application. Fourthly, the heterogeneity source is difficult to be searched. Fifthly, there are multiple TCM syndrome types at post-AMI. In the present meta-analysis, we only analyzed whether an additional use of TCM or TCM preparation can enhance the therapeutic efficacy of Western medicine solely at post-AMI, while the potential function of different TCMs in AMI with the same syndromes was failed to be analyzed. Analyzing the therapeutic effect of TCM preparations containing multiple traditional Chinese herbals could be more complicated or general than that of a single TCM or monomer, which may result in heterogeneity. Moreover, only some of the recruited studies described the detailed chemical components and quality evaluation measures of TCM, which should be further verified (Supplementary Table S3). Finally, several RCTs did not mention the baseline characteristics of subjects and specific drugs used in control groups. As a result, we only analyzed whether TCM or TCM preparations could yield a better efficacy on AMI patients compared with controls, which may result in heterogeneity. The accuracy of the results we obtained needs to be further validated.

## Conclusion

This study concluded that a combination of TCM or TCM preparations with conventional Western medicine can effectively enhance the efficacy on preventing and reversing ventricular remodeling at post-AMI, which also significantly reduces TCMSS, serum levels of BNP and CRP, and the incidence of MACE, as well as improving ventricular remodeling and cardiac function. In addition, no significant differences in adverse events and IVST are detected by additionally applying TCM or TCM preparations on the basis of conventional Western medicine for the treatment of AMI. Our results should be validated in double-blinded, multi-center studies with a large sample in the future because of the relatively high heterogeneity. In addition, in future studies, we will analyze the chemical composition, safety, and efficacy of a series of TCM preparations to further validate their potentials in clinical application.

## Data Availability

The original contributions presented in the study are included in the article/Supplementary Material; further inquiries can be directed to the corresponding author.
