# Supplementary material for: Traditional Chinese Medicine Intervenes Ventricular Remodeling Following Acute Myocardial Infarction: Evidence From 40 Random Controlled Trials With 3,659 Subjects
Source: Front Pharmacol. 2021 Aug 31;12:707394. doi: 10.3389/fphar.2021.707394 (PMC8438202; doi:10.3389/fphar.2021.707394)
Supplement: Supplementary file 1 [file Table4.pdf]

Supplementary Table S4. The results of intervention duration subgroup analysis

Variable	No. of Trials	No. of T	No. of C	SMD (95% CI)	I^2 (%)	*P*
Effectiveness						
2w	2	89	89	3.97 [1.39, 11.32]	0	0.01
4w	3	198	198	6.12 [2.03, 18.50]	55	0.001
6w	3	123	117	3.18 [1.69, 5.95]	0	0.0003
8w	3	80	78	3.32 [1.46, 7.53]	0	0.004
12w	1	25	25	11.29 [1.29, 98.89]	NA	0.03
20w	1	65	65	3.60 [1.22, 10.60]	NA	0.02
24w	4	116	111	5.22 [1.65, 16.59]	0	0.005
TCMSS						
6w	2	92	88	-3.47 [-4.88, -2.06]	0	< 0.00001
8w	2	50	48	-3.25 [-4.97, -1.52]	0	0.0002
12w	1	60	60	-1.10 [-1.78, -0.42]	NA	0.002
20w	1	65	65	-5.04 [-6.38, -3.70]	NA	< 0.00001
24w	2	60	60	-3.28 [-6.07, -0.49]	82	0.02
LVEDD						
2w	2	76	74	-4.59 [-5.33, -3.86]	0	< 0.00001
3w	1	27	23	-2.10 [-4.23, 0.03]	NA	0.05
4w	4	191	188	-3.59 [-6.38, -0.80]	96	0.01
6w	3	95	94	-6.17 [-12.71, 0.36]	96	0.06
8w	5	192	191	-2.16 [-2.99, -1.32]	2	< 0.00001
12w	4	260	260	0.72 [-3.62, 5.06]	96	0.74
16w	1	70	70	0.00 [-0.84, 0.84]	NA	1.00
20w	1	30	30	-3.90 [-5.71, -2.09]	NA	< 0.00001
24w	3	88	88	-2.40 [-4.30, -0.51]	4	0.01
LVESD						
2w	2	70	69	-3.66 [-5.97, -1.36]	76	0.002
4w	1	40	40	-2.70 [-5.94, 0.54]	NA	0.1
8w	3	150	150	-2.11 [-4.67, 0.45]	86	0.11
12w	3	100	100	-2.24 [-8.26, 3.77]	95	0.46
24w	2	51	50	1.57 [-5.44, 8.58]	90	0.66
LVEDV						
2w	2	94	94	-17.08 [-22.77, -11.38]	0	< 0.00001
3w	1	27	23	-2.20 [-8.84, 4.44]	NA	0.52
4w	3	148	148	-2.57 [-20.78, 15.65]	98	0.78
6w	3	138	132	-7.89 [-13.21, -2.57]	0	0.004
8w	1	45	45	0.00 [-3.68, 3.68]	NA	1.00
12w	1	34	34	-10.61 [-17.44, -3.78]	NA	0.002
20w	1	65	65	-11.70 [-20.77, -2.63]	NA	0.01
24w	3	114	108	-14.73 [-34.50, 5.04]	89	0.14
LVESV						
2w	2	94	94	-16.94 [-28.28, -5.59]	87	0.003
3w	1	27	23	-3.10 [-8.68, 2.48]	NA	0.28

4w	3	148	148	-1.71 [-10.98, 7.56]	97	0.72
6w	3	138	132	-9.70 [-12.24, -7.16]	0	< 0.00001
8w	1	45	45	0.00 [-1.78, 1.78]	NA	1.00
12w	1	34	34	-4.39 [-6.34, -2.44]	NA	< 0.00001
20w	1	65	65	-3.05 [-4.31, -1.79]	NA	< 0.00001
24w	1	30	30	-6.02 [-16.34, 4.30]	NA	0.25
LVEDVi						
4w	2	100	100	-4.30 [-7.33, -1.27]	0	0.005
6w	2	71	65	0.37 [-2.69, 3.42]	87	0.81
12w	3	132	131	-6.53 [-7.54, -5.52]	22	< 0.00001
24w	4	310	309	-3.90 [-5.06, -2.74]	94	< 0.00001
LVESVi						
4w	2	100	100	-2.66 [-4.60, -0.72]	0	0.007
6w	2	71	65	-3.17 [-12.58, 6.23]	94	0.51
12w	3	132	131	-4.25 [-6.56, -1.95]	89	0.0003
24w	4	310	309	-4.22 [-10.11, 1.67]	94	0.16
LVEF						
2w	3	134	134	6.00 [0.84, 11.17]	94	0.02
3w	1	27	23	1.40 [-1.51, 4.31]	NA	0.35
4w	7	445	442	5.29 [3.43, 7.16]	80	< 0.00001
6w	5	193	183	5.29 [4.70, 5.88]	0	< 0.00001
8w	5	170	168	5.75 [1.23, 10.27]	94	0.01
12w	9	317	309	4.90 [3.12, 6.68]	71	< 0.00001
20w	1	65	65	18.43 [14.42, 22.44]	NA	< 0.00001
24w	8	448	446	3.82 [1.55, 6.10]	71	0.001

Note: T, TCM group; C, Control group; SMD, standardized mean difference; NA, Not applicable; TCMSS, traditional Chinese medicine syndrome score; LVEDD, left ventricular end-diastolic diameter; LVESD, left ventricular end-systolic diameter; LVEDV, left ventricular end-diastolic volume; LVESV, left ventricular end-systolic volume; LVEDVi, left ventricular end-diastolic volume index; LVESVi, left ventricular end-systolic volume index; LVEF, left ventricular ejection fraction.
