# Supplementary figures and images for: Traditional Chinese Medicine Intervenes Ventricular Remodeling Following Acute Myocardial Infarction: Evidence From 40 Random Controlled Trials With 3,659 Subjects
Source: Front Pharmacol. 2021 Aug 31;12:707394. doi: 10.3389/fphar.2021.707394 (PMC8438202; doi:10.3389/fphar.2021.707394)

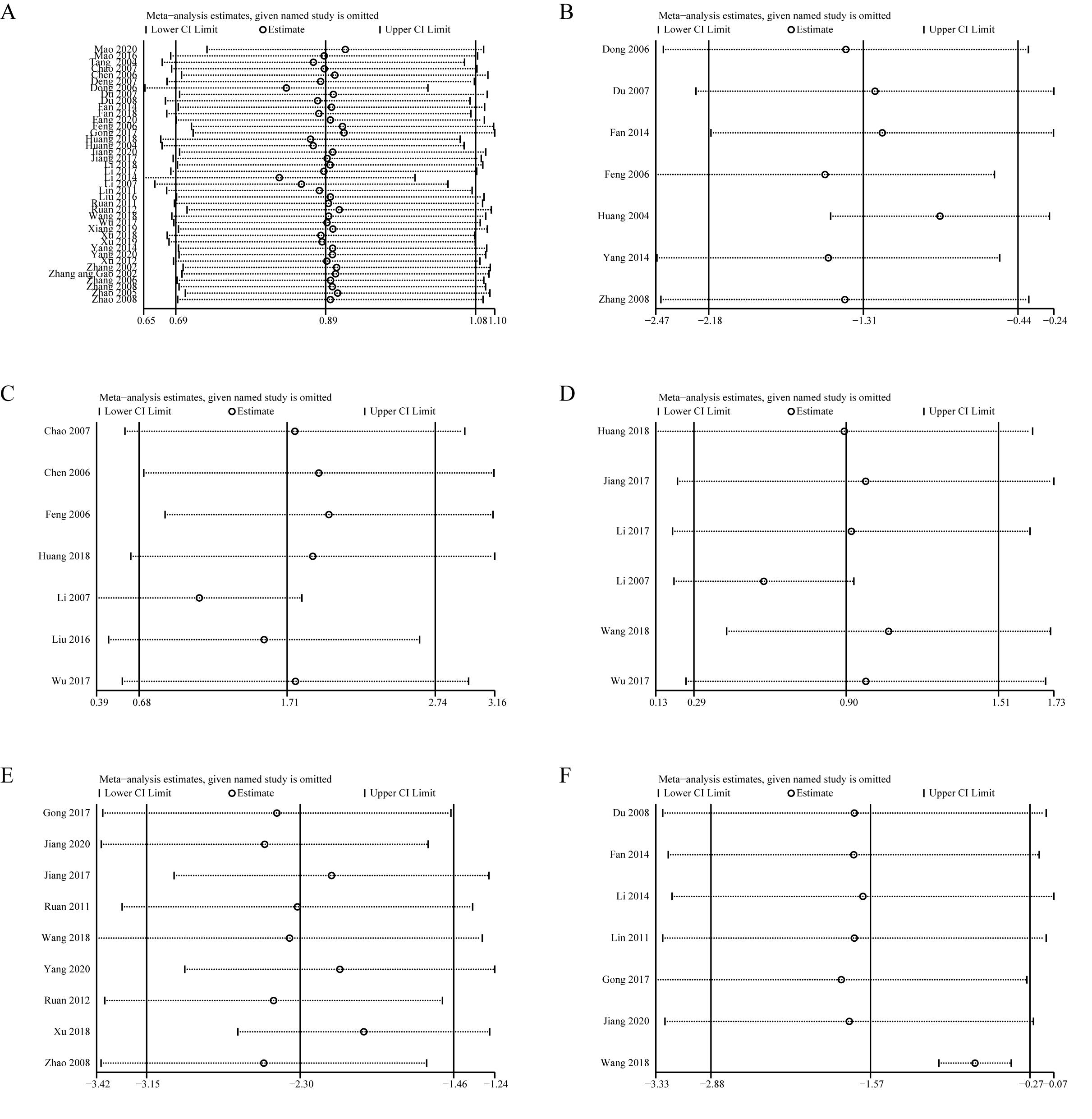

Supplement: Supplementary file 2 [file Image6.TIF]

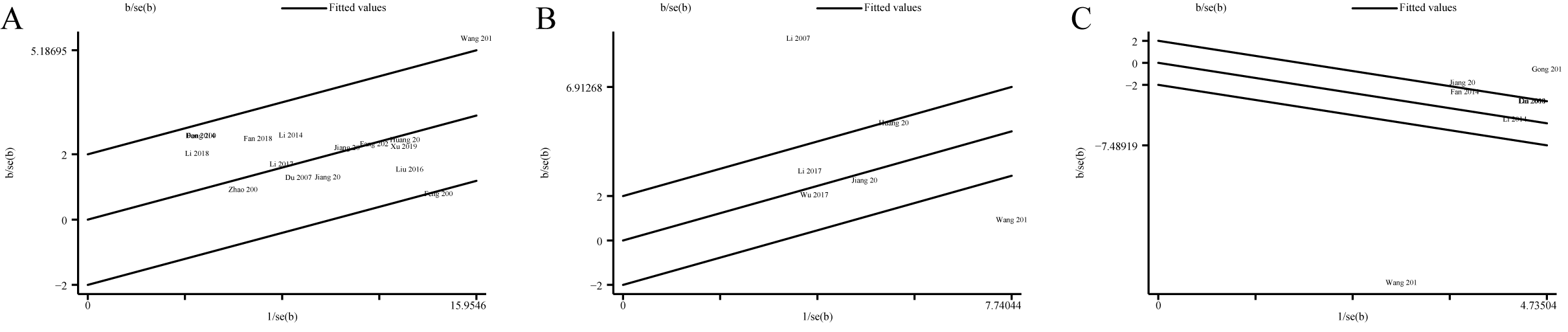

Supplement: Supplementary file 4 [file Image3.TIF]

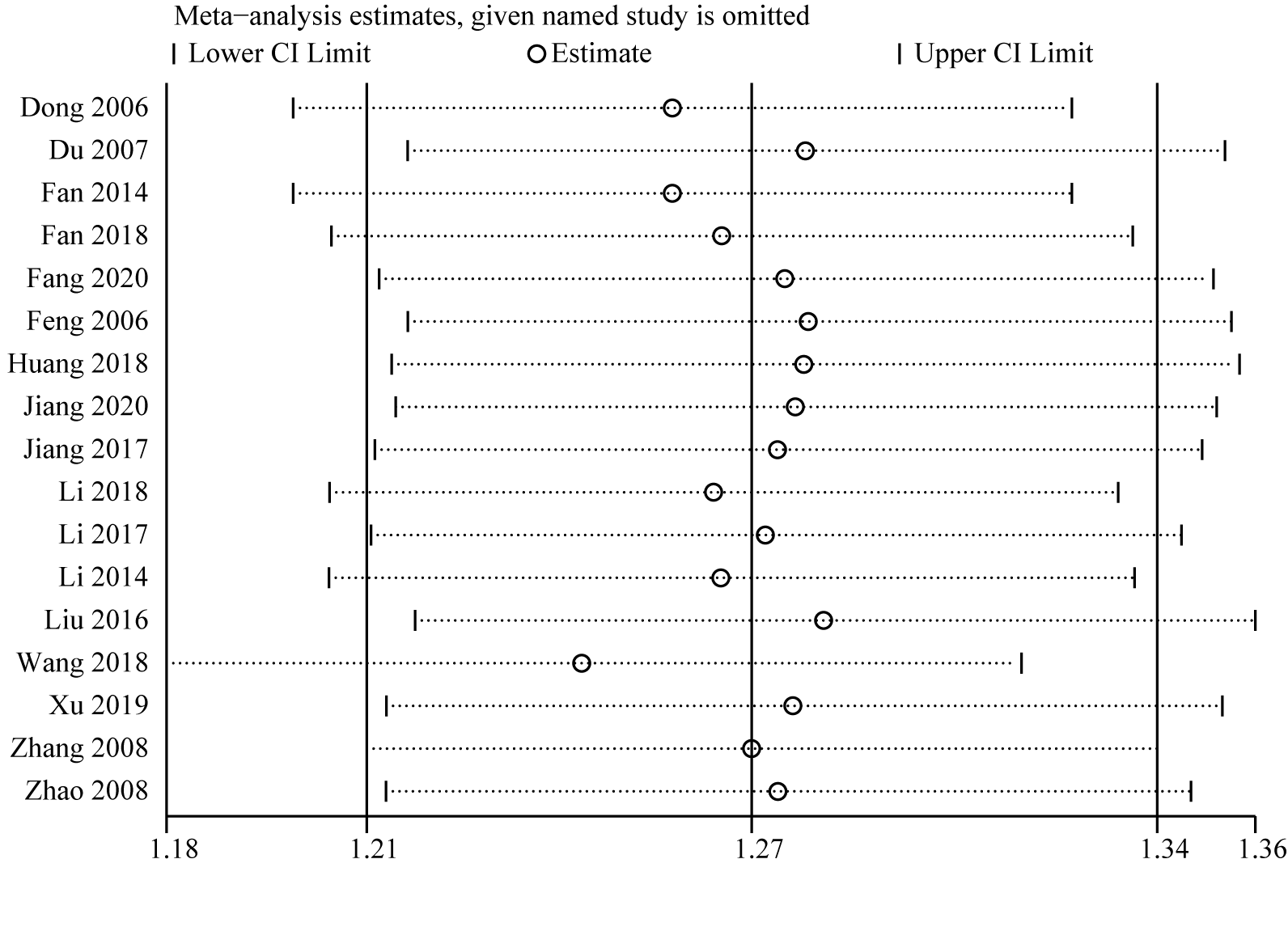

Supplement: Supplementary file 5 [file Image4.TIF]

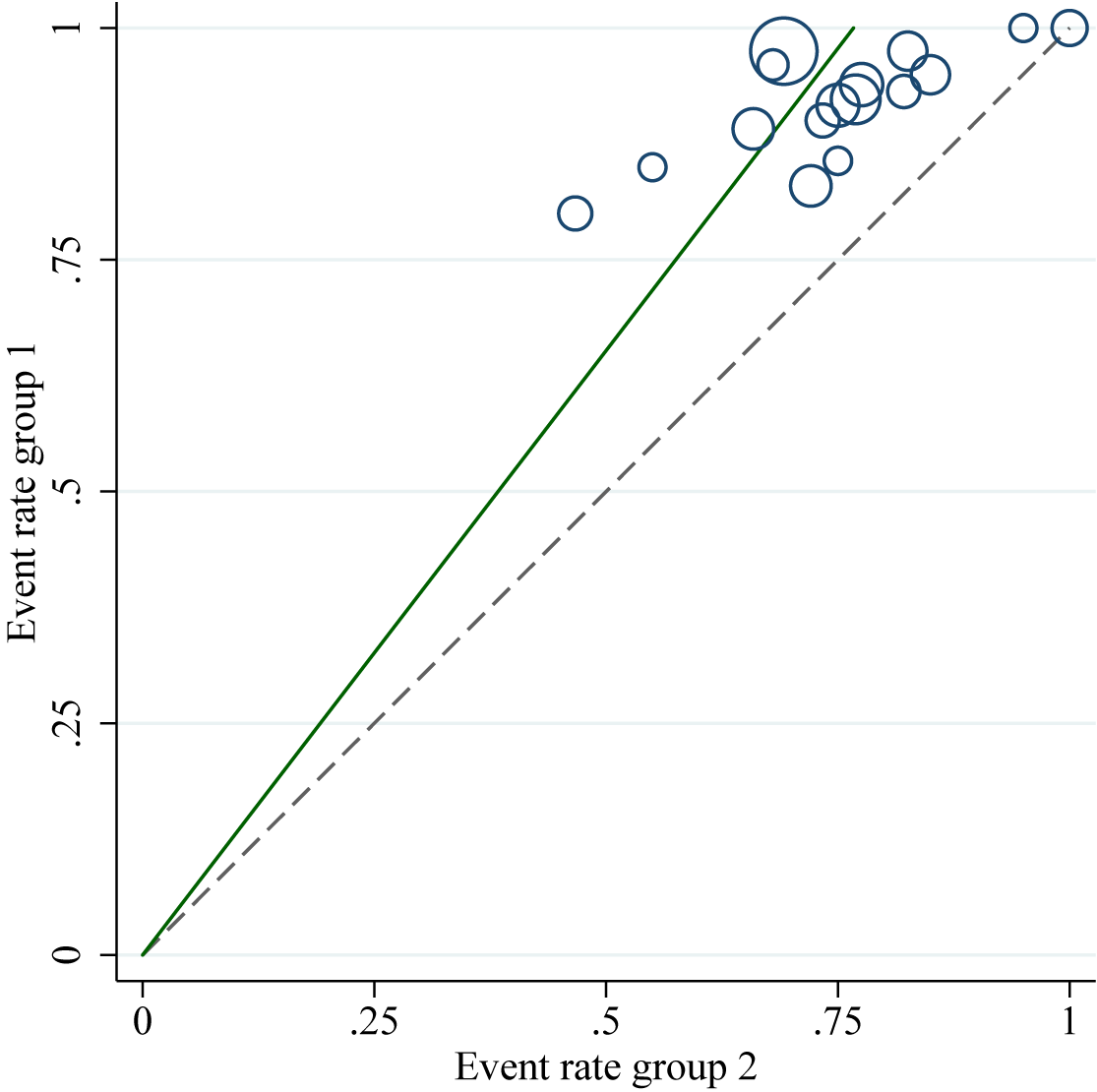

Supplement: Supplementary file 7 [file Image2.TIF]

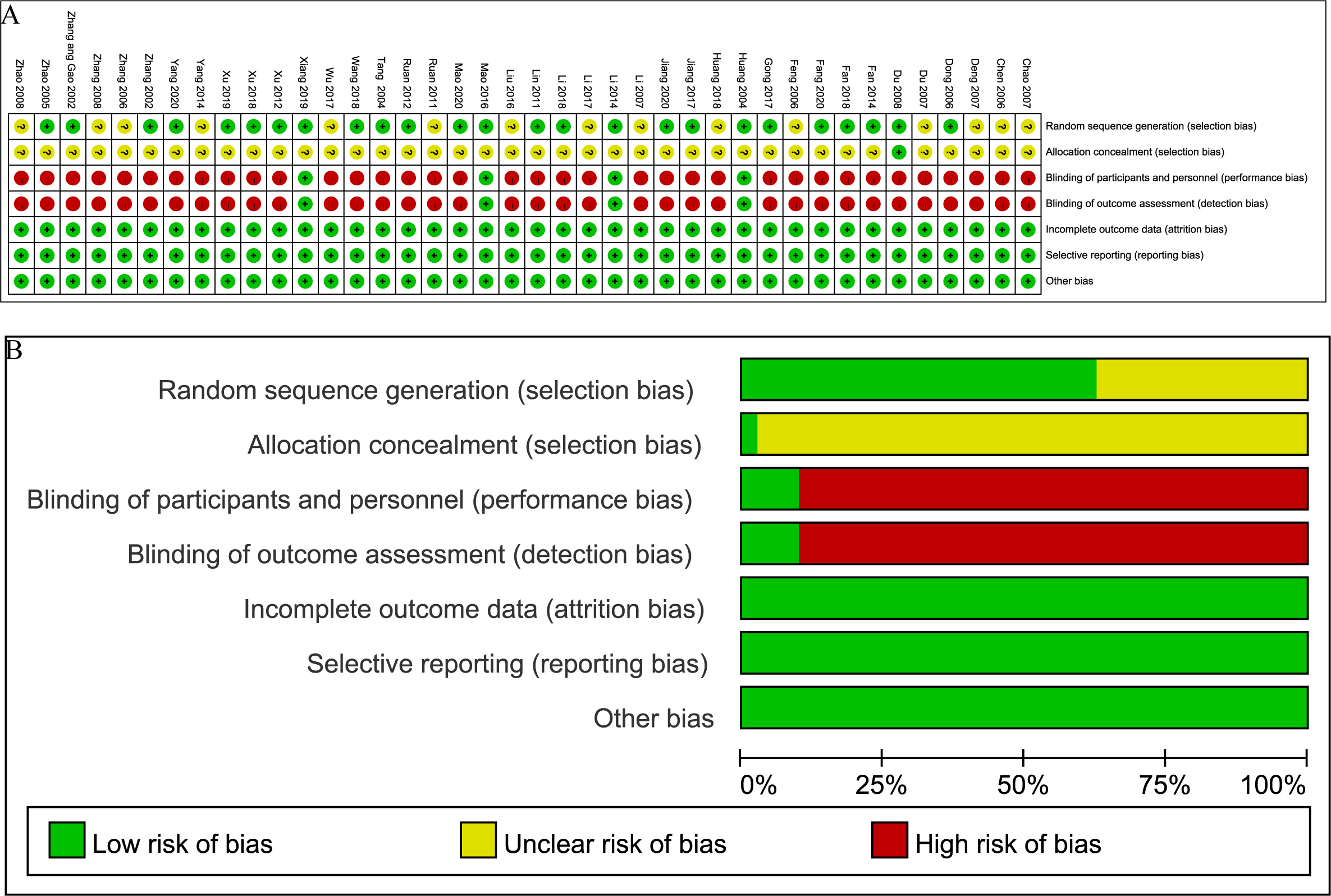

Supplement: Supplementary file 8 [file Image1.TIF]

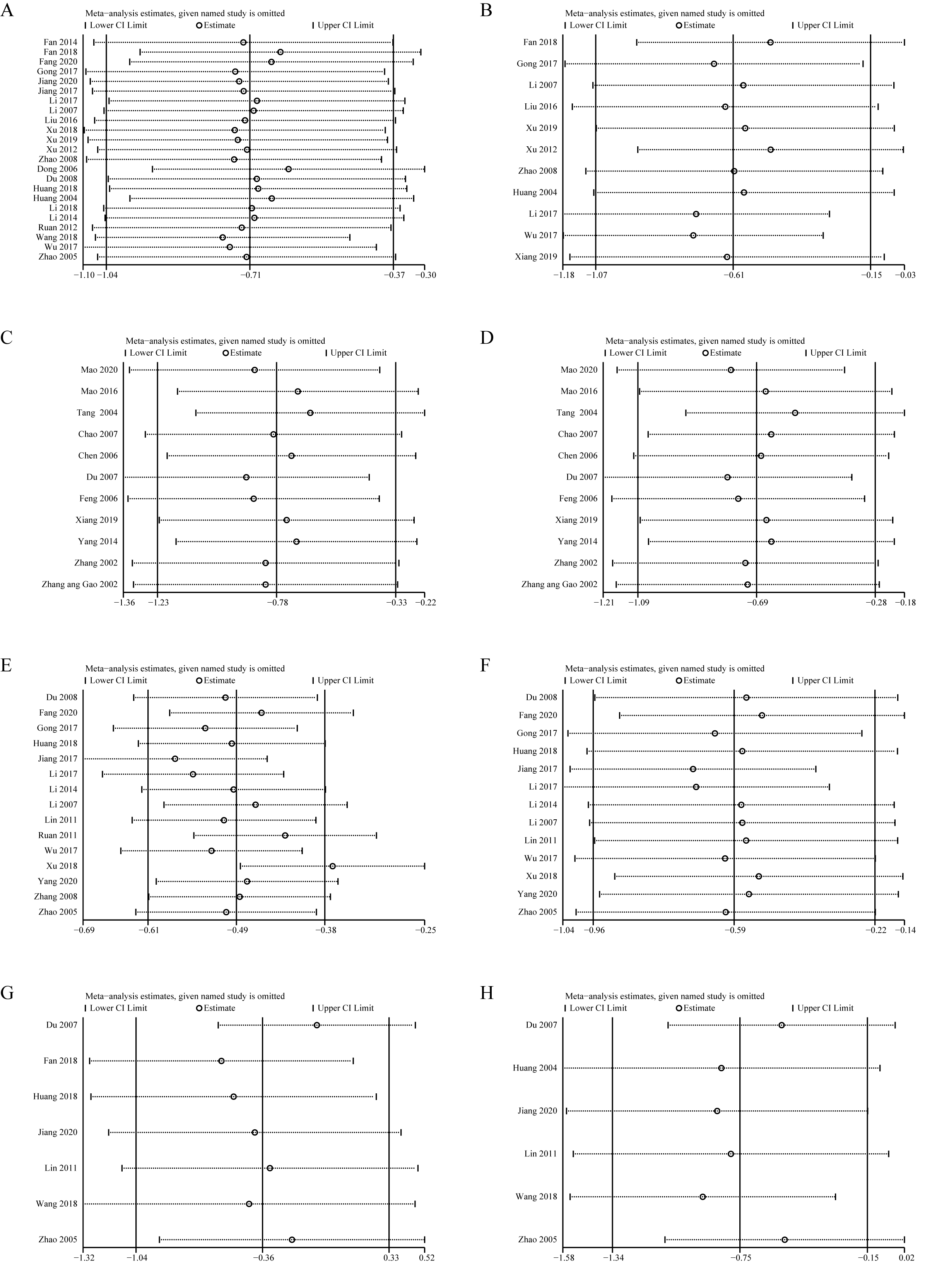

Supplement: Supplementary file 10 [file Image5.TIF]
