# Supplementary material for: Traditional Chinese Medicine Intervenes Ventricular Remodeling Following Acute Myocardial Infarction: Evidence From 40 Random Controlled Trials With 3,659 Subjects
Source: Front Pharmacol. 2021 Aug 31;12:707394. doi: 10.3389/fphar.2021.707394 (PMC8438202; doi:10.3389/fphar.2021.707394)
Supplement: Supplementary file 3 [file Table1.pdf]

Supplementary Table S1. Characteristics of the included studies

Study	Method	Sample size and Age (Years)	Course of treatment	Intervention	Outcomes
Shuai Mao 2020	RCT, non-blind	T: 202, 68.61±8.48 Years<br>C: 201, 67.50±7.82 Years	6 months	T: Tongguan Capsules + standard treatment<br>C: standard treatment	LVESVi, LVEDVi, LVEF; MACE; other clinical assessment indicators
Shuai Mao 2016	RCT, double blind	T: 42, 67.54±8.39 Years<br>C: 41, 68.38±10.41 Years	3 months	T: Danlou tablets + standard treatment<br>C: placebo + standard treatment	LVEDVi, LVESVi, LVEF; MACE; safety assessment
TANG Yao-ping 2004	RCT, non-blind	T: 30, 64 Years<br>C: 30, 65 Years	3 months	T: Xuefu Zhuyu oral liquid + standard treatment<br>C: standard treatment	LDV, LSV, EF
Chao Shengwu 2007	RCT, non-blind	T: 24, 58±18 Years<br>C: 22, 57±11 Years	6 weeks	T: TSN（tanshlnoneII A）injection + standard treatment<br>C: standard treatment	LEDVi, LESVi, LVEF, E/A
Chen Bojun 2006	RCT, non-blind	T: 35, 59.8 Years<br>C: 35, 60.3 Years	6 months	T: Tongguan Capsules + standard treatment<br>C: standard treatment	LVEDVi, LVESVi, E/A, LVEF
Deng Shaolin 2007	RCT, non-blind	T: 77, 61.5±12.8 Years<br>C: 74, 61.4±11.5 Years	4 weeks	T: WenYangBuQi decoction + standard treatment<br>C: standard treatment	6min walk test; LVEF, EDV, ESV, ASL, PSL
Dong Xiaorui 2006	RCT, non-blind	T: 30, 56±9.07 Years<br>C: 30, 57±10.7 Years	8 weeks	T: Yiqihuoxue prescriptione+ standard treatment<br>C: standard treatment	Effective rate; IVST, LVPW, LVDd, WMS; clinical symptoms
Du Lijian 2007	RCT, non-blind	T: 47, 59.8±10.4 Years<br>C: 43, 56.5±8.7 Years	6 weeks	T: Qiangxinling + standard treatment<br>C: standard treatment	Effective rate; AngII, PIIINP, TGF-β1, SOD; LEDVi, LESVi, LVEF, WMSI
Du Wuxun 2008	RCT, non-blind	T: 46, 68.5±8.5 Years<br>C: 44, 69.5±3.5 Years	6 weeks	T: Qishenyiqi Gutta Pill + standard treatment<br>C: standard treatment	WBC, CRP; LVDd, LVEDV, LVESV, LVEF; TCMSS
Fan Yanyan 2014	RCT, non-blind	T: 30, 64.5±12.52 Years<br>C: 30, 61.53±15.41 Years	6 weeks	T: Shenzaoyangxintang+ standard treatment<br>C: standard treatment	Effective rate; LVEDD, IVST, EF, WMSI; CRP
Fan Yuan 2018	RCT, non-blind	T: 25, 69.52±5.55 Years<br>C: 25, 70.55±5.89 Years	3 months	T: Xingengjiuni decoction + standard treatment<br>C: standard treatment	Effective rate; LVEF, LVEDD, LVESD
Fang Keyun 2020	RCT, non-blind	T: 49, 58.08±5.81 Years<br>C: 49, 57.56±5.41 Years	2 weeks	T: Yugeng Tongyu decoction + standard treatment<br>C: standard treatment	Effective rate; LVEF, LVEDV, LVESV, LVEDD; miRNA-21, GDF-15
Feng Qimao 2006	RCT, non-blind	T: 20, 67.15±6.53 Years<br>C: 20, 66.15±6.70 Years	6 months	T: Yiqihuoxue Recipe + standard treatment<br>C: standard treatment	Effective rate; TCMSS, ATII, ALD, ET; LVEDVi, LVESVi, LvmassI, EF, E/A, WMI, WT
Gong Jun 2017	RCT, non-blind	T: 45, 67.2±9.0 Years<br>C: 45, 66.5±10.1 Years	8 weeks	T: Guanxin V Mixture + standard treatment<br>C: standard treatment	NT-proBNP, hs-CRP, MMP-9, AngII, ET-1; LVEDD, LVESD, LVEF, LVESV, LVEDV, LVPWT, IVST

Huang Xianglong 2018	RCT, non-blind	T: 65, 58.80±5.11 Years<br>C: 65, 59.67±4.86 Years	5 months	T: Buyang Huanwu decoction + standard treatment<br>C: standard treatment	Effective rate; LVDd, LVDs, LVPWT, LVM, LVESV, LVEDV, SV, CO, EF, E/A; TCMSS
Huang Xiaoping 2004	RCT, double blind	T: 45, 60.2 Years<br>C: 45, 60.8 Years	8 weeks	T: Yiqi Huoxue capsule + standard treatment<br>C: placebo + standard treatment	IVS, LVPW, LVDd, WMS, LVEF
Jiang Ting 2020	RCT, non-blind	T: 29, 62.83±10.93 Years<br>C: 28, 60.40±10.38 Years	2 months	T: Shenjie Granules+ standard treatment<br>C: standard treatment	Effective rate; LVEF, LVEDD; NYHA; hs-CRP, NT-proBNP; cTnI, CK-MB; TCMSS
Jiang Xiaoxia 2017	RCT, non-blind	T: 48, 50.98±5.86 Years<br>C: 48, 51.27±6.27 Years	4 weeks	T: Xuanbi Tongmai Decoction + Metoprolol + standard treatment<br>C: Metoprolol + standard treatment	Effective rate; TCMSS; LVEF, LVDEE, LVEDV, LVESV, SV, CI; NYHA; cTnI, Nt-proBNP
Li Bo 2018	RCT, non-blind	T: 20, 58.26±6.54 Years<br>C: 20, 59.87±7.54 Years	6 months	T: Shuxuetong injection + standard treatment<br>C: standard treatment	Effective rate; LVDD, LVDS, LVEF; other adverse events
Li Jing 2017	RCT, non-blind	T: 30, 52.88±4.47 Years<br>C: 30, 53.76±4.63 Years	1 month	T: Xuanbi Tongmai Decoction + Captopril + standard treatment<br>C: Captopril + standard treatment	Effective rate; CI, SV, LVESV, LVEDD, LVEF, LVEDV
Li Qiuhong 2014	RCT, double blind	T: 46, 68.6±8.4 Years<br>C: 44, 69.3±3.6 Years	6 weeks	T: Yiqi Huayu Capsule + standard treatment<br>C: placebo + standard treatment	Effective rate; WBC, CRP; LVDd, LVEDV, LVESV, LVEF
Li Zheng 2007	RCT, non-blind	T: 45, 62±15 Years<br>C: 45, 61±14 Years	15 days	T: Guanxinning injection + standard treatment<br>C: standard treatment	LVEF, FS, SV, CO, CI, E/A, LVEDD, LVESD, IVST, LVPWTD, LVEDV, LVESV; death results; MACE
Lin Xiaoduan 2011	RCT, non-blind	T: 46, -<br>C: 44 -	6 weeks	T: Compound Danshen Dripping Pill + standard treatment<br>C: standard treatment	WBC, CRP; LVDB, LVEDV, LVESV, LVEF; TCMSS
Liu Chunxiao 2016	RCT, non-blind	T: 40, 62.5±7.2 Years<br>C: 40, 63.1±6.9 Years	2 weeks	T: Shuxuetong injection + standard treatment<br>C: standard treatment	Effective rate; TCMSS; EPFV, APFV, E/A, FS, LVEF, HR, CO, LVEDd, LVED
Ruan Lei 2011	RCT, non-blind	T: 48, -<br>C: 47, -	6 months	T: Songling Xuemaikang + standard treatment<br>C: standard treatment	LVDEDV, LVEF; BNP
Ruan Xiaofen 2012	RCT, non-blind	T: 28, -<br>C: 28, -	12 weeks	T: Kangxin Decoction + standard treatment<br>C: standard treatment	LVDd, LVEF, IVST, LVPW; BNP
Wang Yunping 2018	RCT, non-blind	T: 120, 58.3±2.6 Years<br>C: 120, 58.6±2.7 Years	1 month	T: Shensong Yangxin capsule + standard treatment<br>C: standard treatment	Effective rate; LAD, EF, LVDd, LVDs, SV, FS; hs-CRP, NT-proBNP, IL-6, NLR; other adverse events

Wu Na 2017	RCT, non-blind	T: 30, 59.21±9.81 Years<br>C: 30, 57.64±10.67 Years	6 months	T: Gualouxiebai Banxia Tang + standard treatment<br>C: standard treatment	LAD, LVDd, LVDs, SV, Sm, FS, E/A, LVEDV, LVESV, LVSV, LVEF
Xiang Zongxing 2019	RCT, double blind	T: 60, 63.0±9.8 Years<br>C: 60, 64.2±11.3 Years	3 months	T: Danlou tablets + standard treatment<br>C: placebo + standard treatment	TCMSS; LVEDVi, LVESVi, LVEF; MACE, other adverse events
Xu Kai 2018	RCT, non-blind	T: 70, 47±11 Years<br>C: 70, 48±9 Years	1 month	T: Shenxiong Injection + Captopril + standard treatment<br>C: Captopril + standard treatment	BNP, LVEDD, LVEDV, LVESV, EF
Xu Xiaojin 2019	RCT, non-blind	T: 40, 56.85±6.71 Years<br>C: 40, 55.92±6.64 Years	6 months	T: Compound Danshen Dripping Pills + Rosuvastatin + standard treatment<br>C: Rosuvastatin + standard treatment	Effective rate; TCMSS; LVEDD, LVESD, LVEF, LVRI; MMP-9, TGF-β1, CTGF
Yang Guoliang 2014	RCT, non-blind	T: 53, -<br>C: 53, -	6 months	T: Heart-protecting musk pill + standard treatment<br>C: standard treatment	LVEDVi, LVESVi, LVEF, LVMI, RWMI
Yang Qi 2020	RCT, non-blind	T: 34, 60.58±6.79 Years<br>C: 34, 60.88±8.41 Years	3 months	T: Gualou Xiebai Banxia decoction + standard treatment<br>C: standard treatment	NT-proBNP, TGF-β, TNF-α, IL-1, MMP-9; LVEF, LVEDV, LVESV
Xu Wei 2012	RCT, non-blind	T: 30, 64±11 Years<br>C: 29, 66±12 Years	3 months	T: Tongxinluo capsule + standard treatment<br>C: standard treatment	LVEF, LVEDD, LVESD; PIIINP
Zhang Jinguo 2002	RCT, non-blind	T: 54, 60.7±8.2 Years<br>C: 54, 61.6±9.1 Years	4 weeks	T: Astragalus lnjection + standard treatment<br>C: standard treatment	LVEDVi, LVESVi, LVEF, ASL, PSL, PER, PFR, TPFR; MDA, CEC, SOD
Zhang Jinguo, Gao Dongsheng 2002	RCT, non-blind	T: 46, 69.2 Years<br>C: 46, 68.9 Years	4 weeks	T: Astragalus lnjection + standard treatment<br>C: standard treatment	LVEDVi, LVESVi, LVEF, ASL, PSL, PER, PFR, TPFR
Zhang Lin 2006	RCT, non-blind	T: 32, -<br>C: 31, -	3 months	T: Yixintong capsule + standard treatment<br>C: standard treatment	LVEDN, LVEF
Zhang Xuezheng 2008	RCT, non-blind	T: 36, 57.08±11.04 Years<br>C: 31, 58.74±11.24 Years	6 months	T: Huixin capsule + standard treatment<br>C: standard treatment	Effective rate; IVST, LVMI, LVEDV, LVEF; TC, TG, LDL-C, HDL-C
Zhao Jing 2005	RCT, non-blind	T: 27, 59.11±11.74 Years<br>C: 23, 63.13±8.29 Years	3 weeks	T: Kaixin capsule + standard treatment<br>C: standard treatment	LVDd, LVEDV, LVESV; AngII, ALD, ET
Zhao Qingfeng 2008	RCT, non-blind	T: 21, 69.28±3.70 Years<br>C: 20, 68.80±2.58 Years	8 weeks	T: Guanxin V Mixture + standard treatment<br>C: standard treatment	Effective rate; TCMSS; BNP; LVEDD, LVESD, LVEF, LVE, LVA

Note: T, TCM group; C, Control group; RCT, randomized controlled trial.
