# Supplementary material for: Traditional Chinese Medicine Intervenes Ventricular Remodeling Following Acute Myocardial Infarction: Evidence From 40 Random Controlled Trials With 3,659 Subjects
Source: Front Pharmacol. 2021 Aug 31;12:707394. doi: 10.3389/fphar.2021.707394 (PMC8438202; doi:10.3389/fphar.2021.707394)
Supplement: Supplementary file 6 [file Table2.pdf]

Supplementary Table S2. Details of conventional Western medication

Study	detailed treatment of conventional Western medication
Mao 2020	Not detailed
Mao 2016	Not detailed
Tang 2004	diazepam, aspirin, isosorbide dinitrate, captopril, bisoprolol
Chao 2007	low molecular heparin, aspirin, nitroglycerin, metoprolol, clopidogrel was added to patients who received PCI
Chen 2006	thrombolytic therapy or PCI+ regular treatment (low molecular heparin, aspirin, nitroglycerin, metoprolol and other symptomatic treatment)
Deng 2007	thrombolytic therapy, Isosorbide mononitrate, low molecular heparin, aspirin, fluvastatin, benazepril, metoprolol
Dong 2006	thrombolytic therapy, antithrombins, antiplatelet agent, nitrates, β-blocker, ACEI
Du 2007	metoprolol, aspirin, verapamil, nitroglycerin
Du 2008	Not detailed
Fan 2014	aspirin, clopidogrel, low molecular heparin, atorvastatin, benazepril, metoprolol
Fan 2018	clopidogrel, aspirin, β-blocker, ACEI, statins, nitrates
Fang 2020	atorvastatin, aspirin
Feng 2006	thrombolytic therapy, β-blocker, diuretic, nitrates, antithrombins, analgesic, lipid-lowering medicine, anti-arrhythmia agent
Gong 2017	aspirin + low molecular heparin
Huang 2018	nitroglycerin, aspirin, atorvastatin
Huang 2004	thrombolytic therapy, low molecular heparin, aspirin, nitroglycerin, potassium magnesium aspartate, atorvastatin
Jiang 2020	aspirin, ticagrelor, low molecular heparin, rosuvastatin, metoprolol, perindopril
Jiang 2017	Heparin, thrombolytic therapy, polarized liquid, metoprolol
Li 2018	Not detailed
Li 2017	nitrates, heparin, aspirin, statins, captopril
Li 2014	aspirin, clopidogrel, low molecular heparin, nitroglycerin (or Isosorbide mononitrate), metoprolol, simvastatin, enalapril
Li 2007	Not detailed
Lin 2011	Not detailed
Liu 2016	thrombolytic therapy, antithrombins, antiplatelet agent, β-blocker, ACEI, CCB, digitalis preparation
Ruan 2011	Not detailed
Ruan 2012	heparin, aspirin, clopidogrel, nitrates, β-blocker, ACEI, statins
Wang 2018	Not detailed
Wu 2017	aspirin, atorvastatin, clopidogrel, metoprolol, ACEI
Xiang 2019	PCI, anticoagulation
Xu 2018	captopril, aspirin, low molecular heparin,
Xu 2019	aspirin, clopidogrel, β-blocker, ACEI, ARB, rosuvastatin
Yang 2014	antithrombins, antiplatelet agent, ACEI, ARB, statins, β-blocker, nitrates
Yang 2020	aspirin, clopidogrel, atorvastatin, ACEI, ARB, β-blocker
Xu 2012	Not detailed

Zhang 2002	nitrates, heparin, aspirin, polarized liquid
Zhang and Gao 2002	nitrates, heparin, aspirin, polarized liquid
Zhang 2006	Not detailed
Zhang 2008	aspirin, clopidogrel, urokinase, amiodarone, nitroglycerin, polarized liquid, low molecular heparin, enalapril, simvastatin, Isosorbide mononitrate, metoprolol
Zhao 2005	nitroglycerin, aspirin, metoprolol, captopril, lipid-lowering medicine
Zhao 2008	ACEI, ARB, nitrates, heparin, aspirin, clopidogrel, β-blocker, lipid-lowering medicine
