# Supplementary material for: Traditional Chinese Medicine Intervenes Ventricular Remodeling Following Acute Myocardial Infarction: Evidence From 40 Random Controlled Trials With 3,659 Subjects
Source: Front Pharmacol. 2021 Aug 31;12:707394. doi: 10.3389/fphar.2021.707394 (PMC8438202; doi:10.3389/fphar.2021.707394)
Supplement: Supplementary file 9 [file Table3.pdf]

Supplementary Table S3. Details of TCM

Study	TCM	Source (Y/N)	Species, concentration	Quality control reported (Y/N)	Chemical analysis reported (Y/N)
Mao 2020	Tongguan Capsules	Y-Guangdong Provincial Hospital of TCM	Radix and rhizome of Salvia miltiorrhiza Bge, Radix of Astragalus membranaceus Fisch. Bunge, Borneol (C10H18O) and Grasshopper	Y- tested by a Drug Safety Committee and approved for the clinical use by the Chinese Pharmacopoeia	Y-HPLC
Mao 2016	Danlou tablets	Y-Jilin Cornell Pharmaceutical Co., Ltd	Radix and rhizome of Salvia miltiorrhiza Bge, Ligusticum chuanxiong, Root of Trichosanthes kirilowii Maxim., Allium macrostemon Bunge, Paeonia lactiflora Pall., Root of Pueraria lobata (Willd.) Ohwi, Alisma plantago-aquatica subsp. orientale (Sam.) Sam., Curcuma phaeocaulis Valeton, Davallia mariesii Moore ex Bak. , Radix of Astragalus membranaceus Fisch. Bunge	Y- Prepared according to Chinese Pharmacopoeia (2015 Edition), approval number: Z20050244	Y-UPLC-MS/MS analysis
TANG Yao-ping 2004	Xuefu Zhuyu oral liquid	Y-Guangdong Zhanjiang Pharmaceutic General Factory	Prunus persica (L.) Batsch (12g), Carthamus tinctorius L. (9g), Angelica sinensis (Oliv.) Diels (9g), Rehmannia chingii H.L. Li (9g), Achyranthes bidentata Blume (9g), Ligusticum chuanxiong (5g), Root of Platycodon grandiflorum (Jacq.) A. DC. (5g), Paeonia lactiflora Pall. (6g), Fruit of Citrus	Y-Prepared according to "Guiding Principle of Clinical Study for New Drug (Chinese drug) in	N

			aurantium L. (6g), Glycyrrhiza uralensis Fisch. (3g), Root of Bupleurum scorzonerifolium Willd. (3g)	Treating Chest Bi"	
Chao 2007	TSN (tanshlnoneII A) injection	N	Extractive from Radix and rhizome of Salvia miltiorrhiza Bge	N	tanshlnoneII A
Chen 2006	Tongguan Capsules	Y-Guangdong Hospital of TCM	Radix of Astragalus membranaceus Fisch. Bunge, Radix and rhizome of Salvia miltiorrhiza Bge., Borneol (C10H18O,), Hirudo medicinalis	Y- Prepared according to Guangdong food and Drug Administration drug standards, approval number: Z03020045	N
Deng 2007	WenYangBuQi decoction	Y-Guangzhou Yifang Pharmaceutical Factory	Aconitum carmichaelii Debeaux (6-10g), Ramulus Cinnamomi(6-15g), Panax ginseng C.A.Mey. (20g), Radix of Astragalus membranaceus Fisch. Bunge (20g), Ophiopogon japonicus (Thunb.) Ker Gawl. (15g), Schisandra chinensis (Turcz.) Baill. (8g), Paeonia lactiflora Pall. (20g), Allium macrostemon Bunge (15g), Trichosanthes kirilowii Maxim. (15g), Glycyrrhiza uralensis Fisch. (6-10g)	N	N
Dong 2006	Yiqihuoxue prescriptione	Y-Henan Hospital of TCM.	Radix of Astragalus membranaceus Fisch. Bunge, Panax ginseng C.A.Mey., Polygonatum sibiricum Delar. ex Redoute, Paeonia lactiflora Pall., arthamus tinctorius L.	N	N
Du 2007	Qiangxinling	Y-Hebei Hospital of TCM	Radix of Astragalus membranaceus Fisch. Bunge (30g), Radix and rhizome of Salvia miltiorrhiza Bge(15g)., Hirudo medicinalis (8g), Root of Pueraria lobata (Willd.) Ohwi (12g), Ligusticum chuanxiong (8g), Paeonia lactiflora Pall. (15g), Angelica sinensis (Oliv.) Diels (20g), Root of Camellia sinensis O.Ktze.(15g)	N	N

Du 2008	Qishenyiqi Gutta Pill	Y-Tianshili Pharmaceutical Group Co., Ltd	Radix of Astragalus membranaceus Fisch. Bunge, Radix and rhizome of Salvia miltiorrhiza Bge., Dalbergia odorifera T. Chen, Panax pseudoginseng var. Notoginseng (Burkill) G. Hoo& C.L. Tseng	Y- Prepared according to SFDA national drug standards YBZ04332003-2008Z, SFDA approval number: Z20030139	N
Fan 2014	Shenzaoyangxintang	Y-Maoming Hospital of TCM in Guangdong Province	Radix of Astragalus membranaceus Fisch. Bunge (20g), Codonopsis affinis Hook.f. & Thomson (20g), Ziziphus jujuba Mill. (20g), Ligusticum chuanxiong (15g), Angelica sinensis (Oliv.) Diels (20g), Ramulus Cinnamomi(10g), Glycyrrhiza uralensis Fisch. (10g), Ziziphus jujuba Mill. (15g), Seed of Platycladus orientalis(L.)Franco(15g), Schisandra chinensis (Turcz.) Baill. (10g), Polygala tenuifolia Willd.(Yuanzhi), Pinellia ternata （Thunb.） Breit. (15g)	N	N
Fan 2018	Xingengjiuni decoction	Y-Jiaozuo Hospital of TCM	Os Draconis (30g), Cornus officinalis Siebold & Zucc. (18g), Talinum paniculatum (Jacq.) Gaertn. (15g), Allium macrostemon Bunge (6g), Angelica sinensis (Oliv.) Diels (18g), Concha Ostreae (30g), Aconitum carmichaelii Debeaux (15g), Dalbergia odorifera T. Chen (6g), Trichosanthes kirilowii Maxim. (12g), Carthamus tinctorius L. (6g)	N	N
Fang 2020	Yugeng Tongyu decoction	Y-Third People's Hospital of Nanyang City	Panax ginseng C.A.Mey. (10-15g), Radix of Astragalus membranaceus Fisch. Bunge (15g), Radix and rhizome of Salvia miltiorrhiza Bge(15g)., Agastache rugosa （Fisch. et Mey.）O. Ktze. (10g), Angelica sinensis (Oliv.) Diels (10g), Corydalis yanhusuo (Y.H.Chou & Chun C.Hsu) W.T.Wang ex Z.Y.Su & C.Y.Wu(10g), Eupatorium fortunei Turcz.(10g), Ligusticum chuanxiong (10g),Citrus reticulata Blanco (10g) ， Pinellia ternata (Thunb.) Breit. (10g),Rheum palmatum L．(6-10g)	N	N
Feng 2006	Yiqihuoxue Recipe	Y-Shuguang Hospital	Radix of Astragalus membranaceus Fisch. Bunge (30g), Pseudostellaria heterophyllaPax ex Pax et Hoffm. (15g), Panax pseudoginseng var.	N	N

			Notoginseng (Burkill) G. Hoo& C.L. Tseng (15g), Sparganium stoloni erum,Buch. -Ham. (15g), Curcuma phaeocaulis Valeton (15g), Prunus persica (L.) Batsch(9g), Carthamus tinctorius L. (9g) , Lumbricus(20g), Cervus nippon Temminck(20g)		
Gong 2017	Guanxin V Mixture	Y-Nanjing Hospital of TCM	Radix and rhizome of Salvia miltiorrhiza Bge., Ligusticum chuanxiong, Carthamus tinctorius L., Paeonia lactiflora Pall., Dalbergia odorifera T. Chen	Y- Prepared according to Drug administration of Jiangsu Province drug standards, approval number: Z04000781	Y-UPLC analysis
Huang 2018	Buyang Huanwu decoction	Y-Zhongshan People's Hospital	Radix of Astragalus membranaceus Fisch. Bunge (25g), Radix and rhizome of Salvia miltiorrhiza Bge (8g)., Carthamus tinctorius L. (8g), Prunus persica (L.) Batsch(8g), Angelica sinensis (Oliv.) Diels (10g), Ligusticum chuanxiong (10g), Lumbricus (6g), Paeonia lactiflora Pall. (15g), Root of Pueraria lobata (Willd.) Ohwi (12g)	N	N
Huang 2004	Yiqi Huoxue capsule	Y-First Affiliated Hospital of Guangzhou University of TCM	Radix of Astragalus membranaceus Fisch. Bunge, Codonopsis affinis Hook.f. & Thomson, Curcuma rcenyujin Y, H. Chenet C.Ling, Ligusticum chuanxiong, Panax pseudoginseng var. Notoginseng (Burkill) G. Hoo& C.L. Tseng, Atractylodes lancea(Thunb.)DC.	N	N
Jiang 2020	Shenjie Granules	Y-Changzhou Hospital of TCM	Panax ginseng C.A.Mey., Daemonorops draco Bl., Paeonia lactiflora Pall., worm medicine	N	N
Jiang 2017	Xuanbi Tongmai Decoction	Y-Changzhou Hospital of TCM	Trichosanthes kirilowii Maxim. (20g), Radix of Astragalus membranaceus Fisch. Bunge (20g), Santalum album L. (20g), Allium macrostemon Bunge (15g), Poria cocos(Schw.)Wolf(15g), Luffa cylindrica (L.) Roem.(15g), Radix and rhizome of Salvia miltiorrhiza Bge(10g)., Panax pseudoginseng var. Notoginseng (Burkill) G. Hoo& C.L. Tseng (10g), Pinellia ternata （Thunb.） Breit. (10g), Angelica sinensis (Oliv.) Diels (10g), Citrus reticulata Blanco	N	N

			(10g), Corydalis yanhusuo (Y.H.Chou & Chun C.Hsu) W.T.Wang ex Z.Y.Su & C.Y.Wu(6g), Panax ginseng C.A.Mey. (6g)		
Li 2018	Shuxuetong injection	Y-Mudanjiang Youbo Pharmaceutical Co., Ltd	Extractive of Hirudo medicinalis (Shuizhi) and Lumbricus	Y- Prepared according to SFDA national drug standards WS3-548(Z-084)-2005(Z), approval number: Z20030139	Hirudin, lumbritin and plasmin
Li 2017	Xuanbi Tongmai Decoction	Y-Yanji Hospital of TCM	Trichosanthes kirilowii Maxim. (25g), Radix and rhizome of Salvia miltiorrhiza Bge(20g), Allium macrostemon Bunge (15g), Carthamus tinctorius L. (10g), Ligusticum chuanxiong (15g), Paeonia lactiflora Pall. (15g), Angelica sinensis (Oliv.) Diels (15g), Root of Aucklandia lappa Decne. (15g), Panax pseudoginseng var. Notoginseng (Burkill) G. Hoo& C.L. Tseng (10g), Panax ginseng C.A.Mey. (8g), Radix of Astragalus membranaceus Fisch. Bunge (20g), Poria cocos(Schw.)Wolf(15g), Corydalis yanhusuo (Y.H.Chou & Chun C.Hsu) W.T.Wang ex Z.Y.Su & C.Y.Wu(10g)	N	N
Li 2014	Yiqi Huayu Capsule	Y-Tangshan Hospital of TCM	Radix of Astragalus membranaceus Fisch. Bunge (60g), Pseudostellaria heterophyllaPax ex Pax et Hoffm. (30g), Ligusticum chuanxiong (15g) Lumbricus(10g), Paeonia lactiflora Pall. (10g), Spatholobus suberectus Dunn (30g)	Y- Prepared according to Drug administration of Hebei Province drug standards, approval number 20050835	N
Li 2007	Guanxinning injection	Y-Shenwei Pharmaceutical Group Co., Ltd	Ligusticum chuanxiong, Radix and rhizome of Salvia miltiorrhiza Bge.	Y- Prepared according to SFDA national drug standards WS3-B-3267-98-2012, approval number: Z13020779	N
Lin	Compound Danshen	Y-Tianshili	Radix and rhizome of Salvia miltiorrhiza Bge., Panax pseudoginseng var.	Y- Prepared according to	N

2011	Dripping Pill	Pharmaceutical Group Co., Ltd	Notoginseng (Burkill) G. Hoo& C.L. Tseng, Borneol (C10H18O)	Chinese Pharmacopoeia (2015 Edition), approval number: Z10950111	
Liu 2016	Shuxuetong injection	Y-Mudanjiang Youbo Pharmaceutical Co., Ltd	Extractive of Hirudo medicinalis and Lumbricus	Y- Prepared according to SFDA national drug standards WS3-548(Z-084)-2005(Z), approval number: Z20010100	Hirudin, lumbritin and plasmin
Ruan 2011	Songling Xuemaikang	Y-Chengdu Kanghong Pharmaceutical Co., Ltd	Root of Pueraria lobata (Willd.) Ohwi, Pinus armandi Franch, Pernulo	Y- Prepared according to Chinese Pharmacopoeia (2015 Edition), approval number: Z10960023	N
Ruan 2012	Kangxin Decoction	Y-Shuguang Hospital	Panax ginseng C.A.Mey., Typha angustifolia L., Radix and rhizome of Salvia miltiorrhiza Bge., Trichosanthes kirilowii Maxim.	N	N
Wang 2018	Shensong Yangxin capsule	Y-Beijing Yiling Pharmaceutical Co., Ltd	Panax ginseng C.A.Mey., Nardostachys jatamansi DC., Ophiopogon japonicus (Thunb.) Ker Gawl., Schisandra chinensis (Turcz.) Baill., Radix and rhizome of Salvia miltiorrhiza Bge., Coptis chinensis Franch., Cornus officinalis Siebold & Zucc., Ziziphus jujuba Mill., Cichlanthus chinensis (DC.) Tiegh., Paeonia lactiflora Pall., Eupolyphaga seusteleophage, Os Draconis	Y- Prepared according to Chinese Pharmacopoeia (2015 Edition), approval number: Z20103032	N
Wu 2017	Gualouxiebai Banxia Tang	Y-Jiangsu Hospital of integrated traditional Chinese and Western Medicine	Fruit of Trichosanthes kirilowii Maxim. (24g), Allium macrostemon Bunge (9g), Pinellia ternata （Thunb.） Breit.(12g), Chinese Baijiu (80ml)	N	N
Xiang 2019	Danlou tablets	Y-Jilin Cornell Pharmaceutical Co.,	Trichosanthes kirilowii Maxim., Allium macrostemon Bunge, Root of Pueraria lobata (Willd.) Ohwi, Ligusticum chuanxiong, Radix and rhizome of Salvia	Y- Prepared according to Chinese Pharmacopoeia	N

		Ltd	miltiorrhiza Bge., Paeonia lactiflora Pall., Alisma plantago-aquatica subsp. orientale (Sam.) Sam., Radix of Astragalus membranaceus Fisch. Bunge, Curcuma rcenyujin Y, H. Chenet C.Ling, Davallia mariesii Moore ex Bak.	(2015 Edition), approval number: Z20050244	
Xu 2018	Shenxiong Injection	Y-Guizhou Yibai injection Pharmaceutical Co., Ltd	Extractive of Radix and rhizome of Salvia miltiorrhiza Bge. And Ligusticum chuanxiong	Y- Prepared according to SFDA national drug standards WS-10001-(HD-1136)-2002-2012-2017, approval number: H52020703	Danshensu, Ligustrazine hydrochloride
Xu 2019	Compound Danshen Dripping Pills	Y-Tianshili Pharmaceutical Group Co., Ltd	Radix and rhizome of Salvia miltiorrhiza Bge., Panax pseudoginseng var. Notoginseng (Burkill) G. Hoo& C.L. Tseng, Borneol ($C_{10}H_{18}O$)	Y- Prepared according to Chinese Pharmacopoeia (2015 Edition), approval number: Z10950111	N
Yang 2014	Heart-protecting musk pill	Y-Shanghai Hehuang Pharmaceutical Co., Ltd	Moschus (Shexiang), Panax ginseng C.A.Mey., Bos taurus domesticus Gmelin, Cinnamomum cassia (L.) J.Presl, Liquidambar orientalis Mill., Bufo bufo gargarizans Cantor, Borneol ($C_{10}H_{18}O$)	Y- Prepared according to Chinese Pharmacopoeia (2015 Edition), approval number: Z31020068	N
Yang 2020	Gualou Xiebai Banxia decoction	Y-Shaoxing People's Hospital	Trichosanthes kirilowii Maxim. (12g), Allium macrostemon Bunge (9g), Pinellia ternata （Thunb.） Breit.(9g), Huangjiu(appropriate amount)	N	N
Xu 2012	Tongxinluo capsule	Y-Shijiazhuang Yiling Pharmaceutical Co., Ltd	Panax ginseng C.A.Mey., Hirudo medicinalis, Buthus martensii Karsch, Paeonia lactiflora Pall., Periostracum cicadae, Eupolyphaga seusteleophage, Scolopendra subspinipes, Santalum album L., Dalbergia odorifera T. Chen, resin of Boswellia carterii Birdw. [B. sacra Fluek.], Ziziphus jujuba Mill., Borneol ($C_{10}H_{18}O$)	Y- Prepared according to Chinese Pharmacopoeia (2015 Edition), approval number: Z19980015	N
Zhang 2002	Astragalus lnjection	Y-Chengdu Dior Jiuhong	Radix of Astragalus membranaceus Fisch. Bunge	Y- Prepared according to SFDA national drug	N

		Pharmaceutical Factory		standards 2001ZFB0171, approval number: Z51021776	
Zhang and Gao 2002	Astragalus lnjection	Y-Chengdu Dior Jiuhong Pharmaceutical Factory	Radix of Astragalus membranaceus Fisch. Bunge	Y- Prepared according to SFDA national drug standards 2001ZFB0171, approval number: Z51021776	N
Zhang 2006	Yixintong capsule	Y-Taian Hospital of TCM	Radix of Astragalus membranaceus Fisch. Bunge, Panax ginseng C.A.Mey., Radix and rhizome of Salvia miltiorrhiza Bge ., Panax pseudoginseng var. Notoginseng (Burkill) G. Hoo& C.L. Tseng, Lumbricus(Dilong), Hirudo medicinalis, Root of Pueraria lobata (Willd.) Ohwi	N	N
Zhang 2008	Huixin capsule	Y-Shandong University of TCM	Radix and rhizome of Salvia miltiorrhiza Bge(30g)., Radix of Astragalus membranaceus Fisch. Bunge (20g), Carthamus tinctorius L. (15g), Prunus persica (L.) Batsch(10g), Panax pseudoginseng var. Notoginseng (Burkill) G. Hoo& C.L. Tseng (10g), Angelica sinensis (Oliv.) Diels (15g), Ligusticum chuanxiong (15g), Hirudo medicinalis (10g), Trichosanthes kirilowii Maxim. (30g), Ramulus Cinnamomi(6g)	Y- Prepared according to Drug administration of Shandong Province drug standards, approval number Z0720030114	N
Zhao 2005	Kaixin capsule	Y-First Affiliated Hospital of Guangzhou University of TCM	Panax quiquefolium L., Radix Astragali, Ophiopogon japonicus (Thunb.) Ker Gawl., Typha angustifolia L. (Puhuang), Ligusticum chuanxiong, Crataegus pinnatifida Bunge, Semen sinapis, Fritillaria thunbergii, Gardenia jasminoides J.Ellis	N	N
Zhao 2008	Guanxin V	Y-Nanjing Hospital of TCM	Codonopsis affinis Hook.f. & Thomson, Ophiopogon japonicus (Thunb.) Ker Gawl., Schisandra chinensis (Turcz.) Baill., Rehmannia chingii H.L. Li, Salvia miltiorrhiza Bge., Paeonia lactiflora Pall.	Y- Prepared according to Drug administration of Jiangsu Province drug standards, approval	Y-UPLC analysis

				number: Z04000781	

SFDA: State Food and Drug Administration, TCM: traditional Chinese medicine
